# Divergence of Arctic shrub growth associated with sea ice decline

**DOI:** 10.1073/pnas.2013311117

**Published:** 2020-12-14

**Authors:** Agata Buchwal, Patrick F. Sullivan, Marc Macias-Fauria, Eric Post, Isla H. Myers-Smith, Julienne C. Stroeve, Daan Blok, Ken D. Tape, Bruce C. Forbes, Pascale Ropars, Esther Lévesque, Bo Elberling, Sandra Angers-Blondin, Joseph S. Boyle, Stéphane Boudreau, Noémie Boulanger-Lapointe, Cassandra Gamm, Martin Hallinger, Grzegorz Rachlewicz, Amanda Young, Pentti Zetterberg, Jeffrey M. Welker

**Affiliations:** ^a^Institute of Geoecology and Geoinformation, Adam Mickiewicz University, 61-680 Poznan, Poland;; ^b^Department of Biological Sciences, University of Alaska Anchorage, Anchorage, AK 99508;; ^c^Environment and Natural Resources Institute, University of Alaska Anchorage, Anchorage, AK 99508;; ^d^School of Geography and the Environment, University of Oxford, OX1 3QY Oxford, United Kingdom;; ^e^Department of Wildlife, Fish, and Conservation Biology, University of California, Davis, CA 95616;; ^f^School of GeoSciences, University of Edinburgh, EH9 3FF Edinburgh, United Kingdom;; ^g^National Snow and Ice Data Center, Cooperative Institute for Research in Environmental Science, University of Colorado, Boulder, CO 80309;; ^h^Centre for Earth Observation Science, University of Manitoba, Winnipeg, MT MB R3T 2N2, Canada;; ^i^Dutch Research Council (NWO), 93460 The Hague, The Netherlands;; ^j^Geophysical Institute, University of Alaska Fairbanks, Fairbanks, AK 99775;; ^k^Arctic Centre, University of Lapland, FI-96101 Rovaniemi, Finland;; ^l^Chaire de Recherche du Canada en Biodiversité Nordique, Université du Québec à Rimouski, QC G5L 3A1 Rimouski, Canada;; ^m^Centre for Indigenous Peoples’ Nutrition and Environment, McGill University, QC H9X 3V9 Montreal, Canada;; ^n^Département des Sciences de l’Environnement et Centre d’Études Nordiques, Université du Québec à Trois-Rivières, QC G8Z 4M3 Trois-Rivières, Canada;; ^o^Center for Permafrost, Department of Geoscience and Natural Resource Management, University of Copenhagen, DK-1350 Copenhagen, Denmark;; ^p^Département de Biologie et Centre d’Etudes Nordiques, Université Laval, QC G1V 0A6 Québec City, Canada;; ^q^Department of Geography, University of British Columbia, Vancouver, BC V6T 1Z2, Canada;; ^r^Biology Department, Swedish Agricultural University, SE-750 07 Uppsala, Sweden;; ^s^Department of Health and Environmental Science, Xi’an Jiaotong-Liverpool University, 215123 Suzhou, China;; ^t^Toolik Field Station, Institute of Arctic Biology, University of Alaska Fairbanks, Fairbanks, AK 99775;; ^u^Department of Geography, Pennsylvania State University, University Park, PA 16802;; ^v^Laboratory of Dendrochronology, School of Forest Sciences, University of Eastern Finland, FI-80101 Joensuu, Finland;; ^w^Department of Ecology and Genetics, University of Oulu and UArctic, 90570 Oulu, Finland

**Keywords:** tundra shrubs, sea ice, Arctic, shrub rings, divergence

## Abstract

Arctic sea ice extent (SIE) is declining at an accelerating rate with a wide range of ecological consequences. However, determining sea ice effects on tundra vegetation remains a challenge. In this study, we examined the universality or lack thereof in tundra shrub growth responses to changes in SIE and summer climate across the Pan-Arctic, taking advantage of 23 tundra shrub-ring chronologies from 19 widely distributed sites (56°N to 83°N). We show a clear divergence in shrub growth responses to SIE that began in the mid-1990s, with 39% of the chronologies showing declines and 57% showing increases in radial growth (decreasers and increasers, respectively). Structural equation models revealed that declining SIE was associated with rising air temperature and precipitation for increasers and with increasingly dry conditions for decreasers. Decreasers tended to be from areas of the Arctic with lower summer precipitation and their growth decline was related to decreases in the standardized precipitation evapotranspiration index. Our findings suggest that moisture limitation, associated with declining SIE, might inhibit the positive effects of warming on shrub growth over a considerable part of the terrestrial Arctic, thereby complicating predictions of vegetation change and future tundra productivity.

## Significance

Two defining features of climate change in the Arctic are the rapid decline of sea ice and “shrubification” of the tundra. While previous studies have inferred warming-related linkages between the two, these have been limited to a few locations. Our Pan-Arctic analysis of shrub growth chronologies reveals two important insights. Tundra shrub growth dynamics are associated with sea ice decline throughout the Arctic; however, while shrubs from most locations increased their growth, more than one-third showed evidence of declining growth in response to warming and drying associated with sea ice loss. These results highlight pronounced growth response heterogeneity across the tundra biome that will have important implications for tundra productivity and vegetation–climate feedback.

Arctic sea ice extent (SIE) is decreasing at an accelerating rate (1–5), with a seasonally ice-free Arctic Ocean expected within a few decades (6). Sea ice decline has elicited major changes in local climates and large-scale atmospheric circulation (7), extending beyond the regions of in situ sea ice changes (8). This includes the impact of winter SIE on upper-level atmospheric flow and subsequent summer air temperature, precipitation, and even soil moisture (9). While this rapid change in the physical system is occurring, the mechanisms by which Arctic sea ice interacts with biological systems are still largely unknown, especially in terrestrial systems (10). For this reason, the study of sea ice effects on Arctic biota has recently been classified as a crisis discipline (2).

The effects of rapidly diminishing SIE on Arctic terrestrial ecosystems, such as changes in shrub growth and tundra productivity, are highly uncertain and understudied at the biome level (2, 3). This is due to i) the complex nature of sea ice dynamics and its strong coupling with atmospheric circulation patterns (7, 11) and climate variables, such as temperature (12), precipitation (13), and humidity (14); 2) the spatial scale of the processes, which are characterized by strong regional variation (15, 16); and 3) the dynamic nature of interannual changes in SIE (1). It is important that we improve understanding of sea ice effects on tundra ecosystems, because changes in the productivity and composition of Arctic vegetation have the potential to amplify or dampen trends in air temperature and sea ice extent through effects on land surface-atmosphere exchanges of carbon and energy (17).

One of the best-documented vegetative responses to Arctic warming is widespread increased productivity and encroachment of deciduous shrubs into lower-statured tundra (18–20). More recently, several studies (21–25) have highlighted the potential for soil moisture to mediate the response of tundra shrub growth to climate warming. A recent synthesis of tundra shrub-ring data showed that shrub growth was more sensitive to interannual climate variability at sites with greater soil moisture (22). Meanwhile, sampling along a moisture gradient within a landscape of northern Alaska revealed a positive correlation between June air temperature and shrub growth at a riparian site and a distinct June air temperature optimum at a drier upland site (23). In Kangerlussuaq, western Greenland, which is a relatively dry area that is experiencing rapid warming, shrub-ring analysis revealed a decline in growth that coincided with decreasing carbon isotope discrimination, low midsummer xylem water potentials, and strong sensitivity of foliar gas exchange to recent rainfall events, suggesting moisture limitation as an underlying cause (25).

Loss of sea ice likely promotes warmer conditions in adjacent terrestrial ecosystems because of the associated dramatic decrease in surface albedo (26). Local warming from sea ice loss can extend several hundred kilometers inland (27–31), and tundra responses to declining sea ice are emerging (10, 32). Examining relationships between sea ice conditions and shrub-ring data throughout the Arctic with explicit consideration of the indirect ways by which sea ice variability can ultimately affect vegetation growth may help assess tundra productivity trajectories. However, few studies have investigated sea ice–shrub growth relationships (33) and none have been conducted at the Pan-Arctic scale. In keeping with widespread observations of increasing shrub abundance, we hypothesized that shrub growth across the tundra biome would be promoted by declining SIE through a positive feedback between declining sea ice and increasing near-surface air temperatures. We expect that one important mechanism through which diminishing sea ice amplifies warming in this context is through greater surface solar absorption in expanding open water areas (34), which leads to local heating and thus favors shrub growth across the tundra biome.

**Fig. 1.**
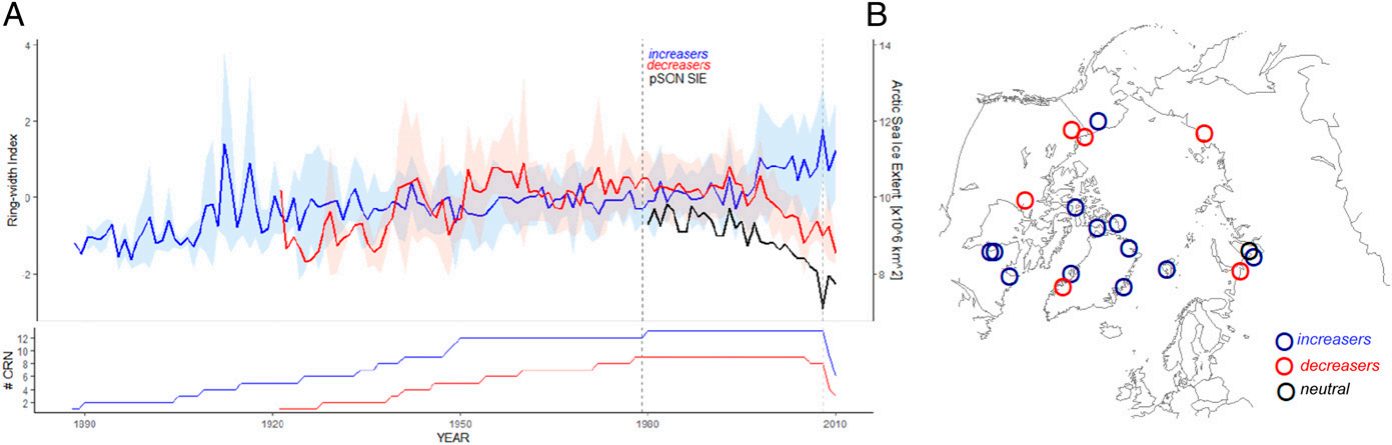
Divergent growth response of Arctic shrubs linked to sea ice decline across the Pan-Arctic region. (*A*) Mean increaser (blue) and decreaser (red) chronologies (RWI with SD) in comparison with seasonal Arctic sea ice extent (black) time series for previous September–October–November (pSON SIE). Vertical dashed lines indicate the common period (1979 to 2008) studied in the synthesis. (*B*) Geographical locations of 23 shrub-ring chronologies and 641 shrubs in total included in the synthesis with indication of sea ice extent–shrub growth response type.

Here we report on tundra shrub growth responses to changes in SIE using 23 annually resolved shrub-ring chronologies of *Betula* and *Salix* shrubs from 19 sites distributed throughout the Arctic from a latitude of 56°N in eastern Canada to 83°N in northern Greenland (Fig. 1*B* and *SI Appendix*, Table S1). In total, we analyzed 641 shrubs with 20,336 growth ring measurements in relation to 1) Pan-Arctic and 2) regional SIE (both monthly and seasonal) and 3) the timing of regional sea ice retreat and freeze-up. Each chronology that was significantly correlated with either Pan-Arctic or regional SIE was aggregated into a specific responder group: increasers, chronologies that were negatively correlated with at least one monthly or seasonal SIE variable; and decreasers, chronologies that were positively correlated with at least one monthly or seasonal SIE variable. In order to test for direct and indirect effects of SIE and summer climate (air temperature, precipitation, and standardized precipitation evapotranspiration index (SPEI)) on shrub growth, we construct piecewise structural equation models (35) (SEMs). We verified the link between each sea ice variable and the growth of individual shrubs by analyzing individual shrub-ring series hierarchically in linear mixed effects (LME) models.

## Results

### Divergent Shrub Growth Responses to Sea Ice.

**Fig. 2.**
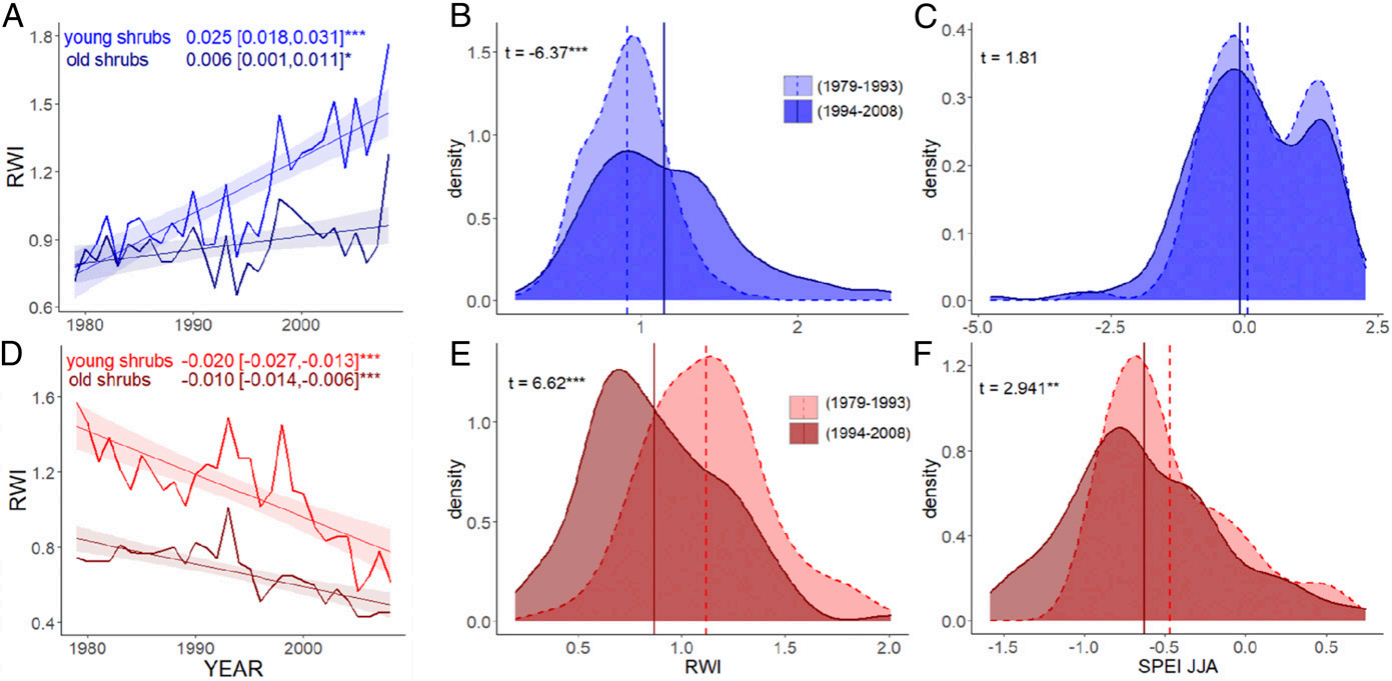
Mean standardized growth pattern for young (i.e., <40 y, light blue line) and old (>39 y, dark blue line) shrubs among (*A*) increasers (363 shrubs, i.e., 46% and 54% young and old shrubs, respectively) and among (*D*) decreasers (251 shrubs, i.e., 61% and 39% young and old shrubs, respectively). Slopes for each time series with associated 95% confidence intervals are indicated on the *Top* with respective colors. Probability density functions for increasers (blue, *B* and *C*) and decreasers (red, *E* and *F*) of mean RWIs and June-to-August SPEI for an early (light colors) and recent (dark colors) period. Mean values are represented by dashed and solid vertical lines for early and recent periods, respectively. Differences between means (Welch’s two-sample *t* test, at 95% confidence level) from early (1979 to 1993) and recent period (1994 to 2008) are reported in the *Top Right*. Please note different scale on the *x* axis between increasers and decreasers. Significant values are indicated with asterisks: *P < 0.05, **P < 0.01, ***P < 0.001.

**Fig. 3.**
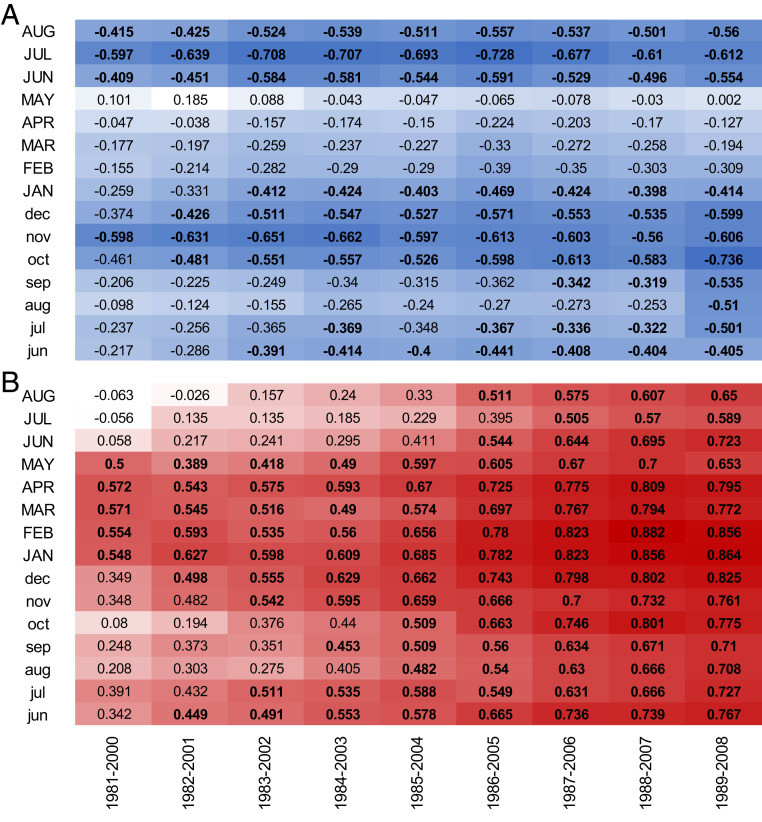
Moving window correlation analyses relating mean increaser and decreaser chronologies to monthly SIE for the Arctic Ocean. (*A*) Increasers (*n* = 13 chronologies; 363 shrubs) and (*B*) decreasers (*n* = 9 chronologies; 251 shrubs) from circumarctic area computed for period 1980 to 2008. The correlations were computed based on 1,000 bootstrapped iterations at a 20-y moving window, offset by 1 y. Significant correlations are marked in bold. Monthly SIE for current (uppercase letters) and previous year (lowercase letters) are indicated on the *Left*.

Although interannual variation in tundra shrub growth was highly correlated with Pan-Arctic SIE throughout the tundra biome (*SI Appendix*, Tables S5–S8), our analyses revealed a strong divergence in the directionality of the association between Pan-Arctic SIE and shrub growth (Fig. 1). While the majority of shrubs displayed increasing growth with declining SIE (increasers, 13 of 23 chronologies; Fig. 2 *A* and *B*), a substantial number of chronologies revealed a significant growth decline (decreasers, 9 of 23 chronologies; Fig. 2 *D* and *E*) (*SI Appendix*, Tables S5–S8). Only one chronology was not significantly correlated with SIE (Fig. 1*B* and *SI Appendix*, Table S1). Furthermore, the strength of correlations between Pan-Arctic SIE and shrub growth increased continually during the period of rapid SIE decline for both increasers and decreasers (Fig. 3).

**Fig. 4.**
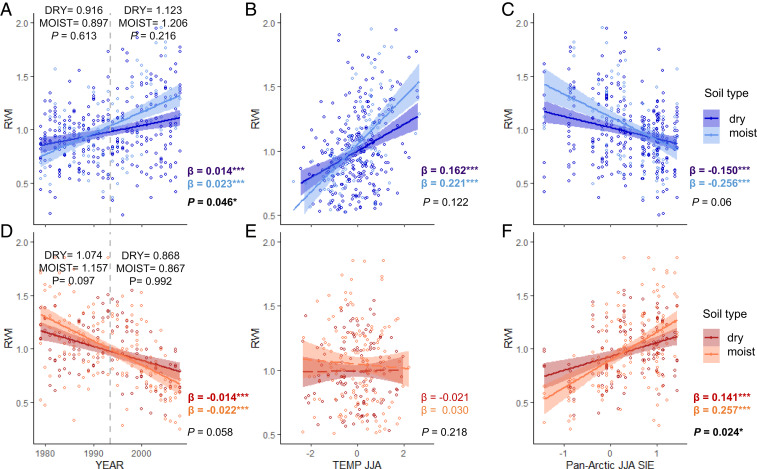
Trends in shrub growth, summer temperature, and sea ice relationships for two groups of responders aggregated per soil moisture type. Standardized annual growth (RWI) of increasers (*A*–*C*; blue) and decreasers (*D*–*F*; red) between dry (dark colors) and moist (light colors) soil moisture types, together with linear relationships (and associated 95% confidence intervals) between shrub chronologies and (*B* and *E*) summer temperature (TEMP JJA) and (*C* and *F*) summer Pan-Arctic sea ice extent (JJA SIE). Nonsignificant trends are marked by dashed lines. Significant levels for differences between the slopes is presented in the *Bottom Right* corner of each plot. Values marked in bold indicate a statistically significant difference (*P* < 0.05). In *A* and *D* plot *P* values from Welch’s two-sample *t* test are reported on the *Top* of the plot together with mean RWI per soil moisture type for early (1979 to 1993) and recent (1994 to 2008) periods. Vertical gray dashed line (*A* and *D*) divides early and recent periods. Linear regression slopes with significant levels (*P < 0.05, ***P < 0.001) for each relationship and entire study period (1979 to 2008) are reported in colored font (dry soil, dark font; moist soil, light font). *N* increasers = 9 (270) and 4 (120) for dry and moist chronologies (mean annual RWI), respectively. *N* decreasers = 4 (120) and 5 (150) for dry and moist chronologies (mean annual RWI), respectively.

**Fig. 5.**
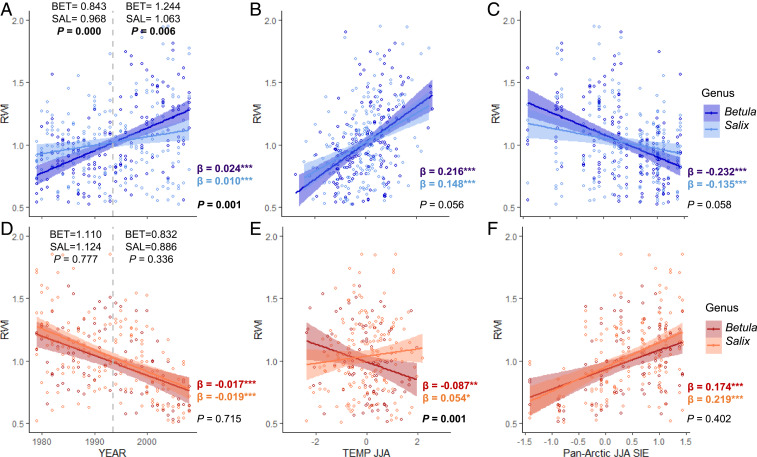
Trends in shrub growth, summer temperature, and sea ice relationships for two groups of responders aggregated per shrub genus. Standardized annual growth (RWI) of increasers (*A*–*C*; blue) and decreasers (*D*–*F*; red) between *Betula* (dark colors) and *Salix* (light colors) chronologies, together with linear relationships (and associated 95% confidence intervals) between shrub chronologies and (*B* and *E*) summer temperature (TEMP JJA) and (*C* and *F*) summer Pan-Arctic sea ice extent (JJA SIE). Significant levels for differences between the slopes are presented in the *Bottom Right* corner of each plot. Values marked in bold indicate a statistically significant difference (*P* < 0.05). In *A* and *D* plot *P* values from Welch’s two-sample *t* test are reported on the *Top* of the plot together with mean RWI per genus type for early (1979 to 1993) and recent (1994 to 2008) periods. Dashed line (*A* and *D*) divides early and recent periods. Linear regression slopes with significant levels (*P < 0.05, **P < 0.01, ***P < 0.001) for each relationship and entire study period (1979–2008) are reported in the *Bottom Left* corner of each plot (*Betula*, dark font; *Salix*, light font). *N* increasers = 6 (180) and 7 (210) for *Betula* and *Salix* chronologies (mean annual RWI), respectively. *N* decreasers = 3 (90) and 6 (180) for *Betula* and *Salix* chronologies (mean annual RWI), respectively.

Increasers showed stronger positive growth trends at moist sites (Fig. 4*A*) and for *Betula* (Fig. 5*A*), whereas negative growth trends for decreasers did not vary significantly across genera (Fig. 5*D*), nor soil moisture class (Fig. 4*D* and *SI Appendix*, Fig. S4). The divergence of growth trends between increasers and decreasers began in the mid-1990s and was stronger among young than among old shrubs (Fig. 2 *A* and *D*). However, divergent growth responses to declining SIE were present across the shrub genera (Fig. 5 *C* and *F* and *SI Appendix*, Fig. S4), age classes (Fig. 2 *A* and *D*), and latitudes studied (*SI Appendix*, Fig. S5). The pattern of divergent shrub growth–sea ice relationships was confirmed when using 1) individual shrubs (instead of chronologies) (*SI Appendix*, Table S12), 2) all shrubs at a site level (*SI Appendix*, Table S13), and 3) when both SIE and shrub-ring data were detrended to retain only interannual variability (*SI Appendix*, Fig. S6 and Table S14).

### Coupling of Regional Sea Ice, Climate, and Shrub Growth.

**Fig. 6.**
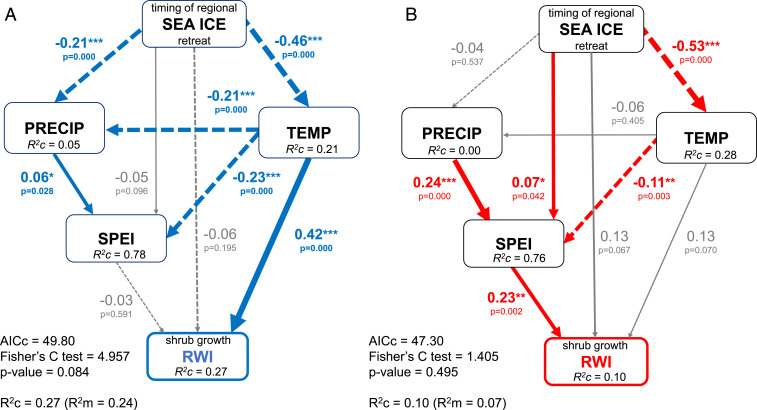
Piecewise SEMs showing the relative importance of the regional sea ice changes and summer climate on mean annual shrub growth for period 1979 to 2008. (*A*) Increasers (blue arrows; *n* = 390 mean annual growth records; 13 chronologies) and (*B*) decreasers (red arrows, *n* = 270 mean annual growth records; 9 chronologies). Each response variable was fit to a linear mixed effect model with random intercept for a site. Positive and negative causal relationships are denoted with solid and dashed arrows, respectively, with the width of the arrows proportional to the strength of the path coefficients. Gray arrows indicate nonsignificant relationships. Numbers on the arrows are standardized parameter estimates with significance levels denoted using asterisks (i.e., *P < 0.05, **P < 0.01, ***P < 0.001). Conditional (R^2^c) and marginal (R^2^m) R^2^ for response variable for each model are indicated in the *Lower Left* corners. Variables used in the model: RWI, ring width index, i.e., standardized mean ring width; timing of regional sea ice retreat (LRD, last retreat day of sea ice expressed as day of the year for each sea ice region, z-score; see Materials and Methods); summer (i.e., June to August) standardized precipitation evapotranspiration index (SPEI), precipitation (PRECIP), and temperature (TEMP). Full model statistics with SEs are presented in *SI Appendix*, Tables S10 and S11.

Having identified divergent directionalities in shrub growth responses to declining SIE, we next applied SEMs in separate analyses of increasers and decreasers. These models, conducted at the regional SIE level, revealed that warmer air temperature favored growth of increasers, while limiting growth of decreasers through changes in climatic water balance (i.e., lower SPEI) (Fig. 6). For increasers, declining regional SIE was associated with increasing local air temperature and increasing precipitation, which was associated with greater shrub growth (*SI Appendix*, Table S10). For decreasers, declining SIE was associated with increasing local temperature and declining SPEI, which is indicative of increasingly dry conditions that may have limited shrub growth (*SI Appendix*, Table S11). Specifically, earlier retreat of regional sea ice for increasers (Fig. 6*A*) was associated with both increased summer temperature (β = −0.46, SE = 0.05, df = 376, *P* < 0.01) and increased summer precipitation (β = −0.21, SE = 0.06, df = 375, *P* < 0.01). For decreasers, earlier retreat of regional sea ice (Fig. 6*B*) was also significantly related with higher current June–August temperature (β = −0.53, SE = 0.06, df = 255, *P* < 0.01), but it was associated with lower current June–August SPEI (β = 0.07, SE = 0.02, df = 255, *P* = 0.04), with no significant impact on summer precipitation (β = −0.04, SE = 0.08, df = 259, *P* = 0.54). Growth of increasers was significantly and positively related to temperature (β = 0.42, SE = 0.02, df = 370, *P* < 0.01). Meanwhile, growth for decreasers was positively related to summer SPEI (β = 0.23, SE = 0.05, df = 255, *P* < 0.01) and not to temperature (β = 0.13, SE = 0.02, df = 255, *P* = 0.07). Results for decreasers suggest that moisture limitation associated with declining SIE may be a significant controlling factor for tundra shrub growth in some arctic locations.

## Discussion

Tundra shrub growth trajectories exhibited a pronounced and increasing lack of uniformity during the period of declining SIE. This is largely explained by the divergence of growth responses to warming trends between drier and wetter sites. Despite the dichotomy in directional responses to declining SIE, tundra shrub sensitivity to sea ice loss was nearly ubiquitous, because all but one chronology bore a significant relationship with SIE. Shrub growth responses clearly diverged into two groups: those showing increases and those showing decreases in growth during the period of SIE decline. SEM allowed for a mechanistic interpretation of the coupling between sea ice dynamics, regional climate, and shrub growth in the Pan-Arctic. Shrub growth sensitivity to declining regional SIE was mediated by variations in regional summer climate (i.e., temperature and moisture availability, Fig. 6). Rising air temperature was widespread across all sampling sites (*SI Appendix*, Table S4), but shrub growth may have responded positively only at sites where moisture was not or did not become colimiting.

The two shrub growth responder groups demonstrated different associations with climate variables. For increasers, declining SIE was associated with increasing precipitation, dampening the potential for moisture limitation of shrub growth (Fig. 6*A*). For decreasers, declining regional sea ice and rising temperature were associated with declining SPEI, suggesting that increasing evaporative demand was not compensated by greater precipitation, thereby creating the potential for moisture limitation. Moreover, in all SEMs constructed for decreasers and regional sea ice, the relationships between sea ice, SPEI, and shrub growth always outweighed the positive impact of temperature on shrub growth (*SI Appendix*, Table S11), which was not the case for increasers. Sites with decreaser shrub populations were inherently drier (i.e., lower recent summer SPEI and lower summer precipitation) than sites with increasers (Fig. 2 *C* and *F* and *SI Appendix*, Fig. S7 and Table S4). In fact, the greatest response to declining SIE among decreasers was found for habitats classified as moist (Fig. 4*F* and *SI Appendix*, Fig. S4*H*), where shrubs may be less well adapted to low moisture availability. While moisture availability has long been thought to indirectly affect tundra productivity via soil nutrient availability (36), evidence of drought stress in arctic tundra plants has historically been limited (37). As declines in tundra “greenness” are becoming evident in some areas of the Arctic (38–40), moisture limitation of shrub radial growth in the warming Arctic is becoming increasingly apparent (21–25). Our results suggest this shift in limiting factors for tundra shrub growth may be coupled with sea ice dynamics (Fig. 6*B*).

SEMs for decreasers that utilized Pan-Arctic sea ice data explained substantially greater variance than SEMs using regional SIE (*SI Appendix*, Table S11). Additionally, all Pan-Arctic SEMs for both responders manifested strong and direct links between seasonal Pan-Arctic sea ice and shrub growth (*SI Appendix*, Tables S10 and S11), which indicate variance explained by Pan-Arctic SIE in shrub growth series and not accounted for by local climate data. This finding of stronger Pan-Arctic than regional SIE relationships might reflect “a common forcing” [sensu Macias-Fauria et al. (31)] in which Pan-Arctic SIE influences weather patterns, such as cyclonic activity, and perhaps even large-scale atmospheric circulation patterns, such as Arctic and North Atlantic Oscillations (AO, NAO). These changes might, in turn, impact tundra shrub growth through changes in cloudiness, humidity (41, 42), or timing of snow melt (43). This, and other potential mechanisms of synoptic forcing constitute an important knowledge gap that requires further research and acquisition of high-resolution in situ data.

Important questions moving forward should focus on the mechanisms by which moisture availability may be affecting tundra productivity (i.e., direct vs. indirect via variables such as soil nutrient availability) and how changes in precipitation predicted for the Arctic (44) may or may not counteract the effect of rising air temperature on evaporative demand and moisture availability to tundra shrubs. Specifically, variation in microclimate associated with changes in active layer depth (45), changes in the depth and/or duration of snow cover (43, 46), and rain-on-snow events (47, 48) have the potential to further modify shrub growth responses to changes in SIE and climate. Moreover, biotic factors, such as herbivory, have the potential to influence shrub growth responses to changes in climate (49). Also, it is likely that sampling at higher spatial density would reveal that increasers and decreasers can often co-occur within the same landscape (23), provided that the regional climate is dry enough to induce moisture limitation for the most drought intolerant species in the driest habitats. It is important to note that main stem radial growth is just one of many aspects of shrub productivity. Relationships among stem radial growth and other aspects of growth and reproduction represent important priorities for future research. The spatial scale of Arctic shrub growth divergence requires further examination, and future studies should strive to investigate sea ice–shrub growth responses along moisture gradients within a given landscape. Arctic shrub-ring studies would also benefit from integrating experimental manipulations of moisture availability with detailed microclimate and physiological measurements of plant–water relations to corroborate the presence of moisture-limited growth at the levels of species and habitat.

Our results demonstrate that declining regional sea ice and coupled changes in regional summer climate are driving divergent shrub growth trajectories across the Arctic. The temporal resolution of our analyses allowed us to reveal that growth of increasers and decreasers was relatively similar (both in direction and magnitude) prior to the point of divergence in the mid-1990s (Fig. 1*A*), suggesting more coherent shrub growth across the Arctic before the recent onset of dramatic sea ice decline (5). This finding relates to the well-known phenomenon of divergent tree growth responses to warming in the boreal forest (50, 51). While divergence between tree growth and mean summer temperatures in the boreal zone generally began around 1960, the shrub growth divergence that we have identified began later, near the end of the 20th century. We hypothesize that divergent woody vegetation growth responses to climate warming have been spreading north as the magnitude of climate warming increases and the geography of growth-limiting factors, favoring precipitation over temperature, shifts northward (52). Thorough assessment of these phenomena at the tundra biome level will require a broader dataset of shrub-ring chronologies covering both longer timespans and the most recent decade.

The implications of increasing heterogeneity of Arctic shrub growth trajectories to sea ice-induced changes in regional climate might be widespread, including permafrost degradation and heat flux alteration in response to changes in vegetation cover and albedo (45), increased wildfire risk (53), and susceptibility to insect outbreaks (54). Furthermore, the declining growth response revealed for decreasers might be indicative of more limited woody biomass production and reduced carbon sequestration in a pool (i.e., wood) with a long mean residence time. Conversely, the enhanced growth response with warming in the increasers suggests potential for a strengthening of the carbon sink in some areas of the Arctic. Changes in tundra shrub productivity, in either direction, may also alter habitat and forage quality for a wide range of Arctic herbivores (32, 47), some of which are experiencing pronounced population declines (55)_,_ while others are showing northward range expansion (56). Our finding that sea ice decline and associated changes in regional climate are driving divergent shrub growth responses not only emphasizes the important role of teleconnections in tundra ecosystem function, but adds an extra layer of complexity to projections of future tundra carbon cycling. Specifically, the potential for SIE-induced changes in moisture availability to drive tundra shrub radial growth declines must be accounted for in order to refine projections of future climate change feedback in the Arctic region and beyond.

## Materials and Methods

### Shrub-Ring Chronologies.

We acquired both published and unpublished deciduous shrub-ring chronologies that were distributed throughout the Arctic region [*SI Appendix*, Table S1, and the Dryad Digital Repository: https://doi.org/10.5061/dryad.kh1893248 (57)] and covered, if possible, the entire 40-y-long period of passive microwave satellite-based estimates of Arctic SIE (1979 to the present). In order to perform a comparable study at the biome level, our synthesis focused on two shrub genera of commonly studied (21–25, 33, 45, 48, 54) and widespread deciduous shrubs: *Betula* and *Salix*. We analyzed both shrub-ring chronologies and shrub-ring series from individual shrubs. Building chronologies at the level of site and species afforded the opportunity to examine the quality of the data, while also providing a summary of shrub growth at the level of site and species.

All shrub-ring series (i.e., raw measurements of annual ring widths after cross-dating for each individual sample) submitted by the contributors were subjected to cross-dating quality checks. Standard dendrochronological statistical tests, including 1) the expressed population signal (58) (EPS), 2) mean sensitivity (MS), 3) first-order autocorrelation [AR(1)] and mean pairwise correlation between all cross-sections (rbar.tot) were performed using the dplR package (59, 60) in R version 3.5.1 (61) (*SI Appendix*, Table S1). Shrub-ring data were included in our analyses if the corresponding chronologies 1) covered the common period (1979 to 2008) and 2) had an EPS [a theoretical indicator of how well the chronology represents the population mean (58)] greater than 0.75 (*SI Appendix*, Table S1). Among the 32 submitted chronologies, 9 were excluded because they failed to pass our threshold for minimum chronology coherence (i.e., EPS < 0.75 for detrended series) and/or because of biased sampling design. Additionally, sites were classified as either moist or dry (*SI Appendix*, Table S1) according to the protocol proposed by Myers-Smith et al. (22). Moist and wet soil classes were combined into the moist soil type. The dry soil moisture type was assigned when, during the warmest month of the year, the top 2 cm of the soil were dry to the touch (more than one inspection was preferred). The moist soil moisture type was assigned when surface soils were moist throughout the growing season or standing water was present during the warmest month.

Our final dataset consisted of 23 chronologies (9 *Betula* spp. chronologies and 14 *Salix* spp. chronologies), 641 shrubs (306 *Betula* shrubs and 335 *Salix* shrubs), and 753 cross-sections (*SI Appendix*, Table S1 and Fig. S2). In total, 20,336 growth rings (23 chronologies, 641 shrubs) were analyzed initially (Pearson’s correlations and linear regressions) for the period 1979 to 2008. On average, 33 cross-sections (min = 15, max = 77) were included in each chronology. Although some shrubs were subjected to serial sectioning (62), only root collar–stem base cross-sections (after cross-dating) were used in chronology construction. This approach was applied because previous studies have shown that ring widths at these basal locations are most sensitive to climate (63) and because many of the studies in the synthesis only collected discs at this location. Differences in mean standardized shrub growth for chronologies and for selected climatic variables (Fig. 2 *B*, *C*, *E*, and *F* and *SI Appendix*, Tables S3 and S4) were compared between an early (1979 to 1993) and recent (1994 to 2008) period, i.e., by splitting the entire study period (1979 to 2008) into two equal halves of 15 y. Differences in means for these two time periods were compared using Welch’s two-sample *t* test.

### Detrending.

All analyses were performed using standardized chronologies. Shrub-ring data were detrended using the diameter regional curve standardization (dRCS) (64, 65), an approach designed to remove the effects of stem diameter on ring width while preserving long-term trends in shrub radial growth (see below). In the first step of dRCS detrending, we analyzed the effect of stem radius/radial diameter on ring widths (*SI Appendix*, Figs. S8 and S9). For the majority of shrubs we observed a positive effect of stem diameter on ring width (i.e., an increase in ring widths with an increasing radius). Ring width indices (RWIs) were calculated as ratios of observed-to-expected growth and site-level indices were processed to produce signal-free chronologies (66). Site-level and responder type (Fig. 1*A* and *SI Appendix*, Fig. S1) chronologies were computed as the arithmetic mean of RWI. Mean correlation between chronologies (1979 to 2008) was 0.108 and 0.4 for increasers and decreasers, respectively.

Shrub-ring standardization was performed in CRUST (65) and initially several detrending methods were tested (i.e., modified negative exponential, splines of various rigidity, dRCS, and age regional curve standardization [aRCS]). We aimed to standardize all of the shrub-ring data using the same method to avoid potential detrending bias across species and sites. The goal of standardization was to eliminate temporal variation in ring widths that can be attributed to the age or size of the shrub stem at the time that the ring was formed, while preserving low-frequency trends in shrub radial growth that could be influenced by the sea ice decline and associated changes in climate. Standardization also normalizes ring width magnitude across species and sites, allowing for aggregation of shrub-ring chronologies. After testing a variety of common growth ring data standardization methods, we settled on dRCS as the most suitable method for both theoretical and statistical reasons. Most detrending methods are applied at the level of the individual series with the aim of removing low-frequency variation (decadal-to-century scale trends), while retaining high-frequency variation (interannual variability). The low-frequency variation that is removed in this process may be derived from both intrinsic (e.g., age, size, canopy position) and extrinsic sources (e.g., climate trends). In recent years, the RCS method has gained prominence for its potential to remove the low-frequency trends derived from intrinsic sources, while retaining low-frequency trends driven by extrinsic forces, such as trends in climate (67). Briefly, RCS detrending involves 1) aggregating individual tree- or shrub-ring series that vary strongly in age and size, 2) aligning the series by age or size to define the overall effect of age or size on the ring widths (typically a spline curve), 3) removing the empirically derived age or size effect from each series through calculation of ratios or residuals of observed-to-expected growth for an individual of a given age or size. While RCS detrending is appealing as a method to examine low-frequency trends in tree or shrub growth, a number of pitfalls have been identified (68). One of the more challenging of these pitfalls is known as the “modern sample bias,” which emerges when a single age-related detrending curve is fit to a heterogeneous sample of both fast and slow growing trees or shrubs. The fast-growing individuals tend to be younger, while the slow-growing individuals tend to be older. Errors in the fit of the single detrending curve to these contrasting groups can lead to spurious trends in the resulting tree- or shrub-ring chronology. In an effort to mitigate the modern sample bias, Briffa and Melvin (68) advocated for the use of multiple curve RCS, in which separate detrending curves are fitted to fast- and slow-growing individuals. An important constraint of multiple curve RCS is that it requires a larger sample size than single curve RCS (∼50 series/curve) (65).

Development of a shrub-ring chronology is generally much more labor intensive than development of a tree-ring chronology, due to application of serial sectioning (62) and staining of thin sections (69) versus sanding of tree increment cores. For that reason, shrub-ring chronologies are generally constructed using fewer samples than tree-ring chronologies and shrub-ring chronologies that can support multiple curve RCS are rare. The dRCS method (64, 65) is a less well-known variant of RCS, in which the detrending curve is defined by the empirical relationship between ring widths and the radius of the stem at the time the ring was formed. Replacing age with radius is a means to address the more rapid decline in ring width with age in fast-growing individuals and the more gradual decline in ring width with age in slow-growing individuals. For this reason, we argue that dRCS may be the preferred standardization method when sample sizes are too small to support multiple curve RCS and when the aim of the study is to examine low-frequency growth trends that are driven by extrinsic forces (25, 70). In our shrub-ring dataset, we generally found weak trends between ring widths and the size or the age of the stem, meaning that standardization generally had a small effect on the resulting shrub-ring chronology, as observed in other shrub-ring studies (25).

The robustness of the relationship between sea ice and shrub growth was verified using so called “double detrended” time series. For this test, dRCS ring width indices and raw Pan-Arctic SIE time series were detrended using autoregressive (AR) modeling (71). Doing so, we aimed to examine high frequency variation, where interannual variability in shrub growth was related to detrended (using the same method) seasonal SIE time series. Other detrending methods were tested, including modified negative exponential curve and cubic smoothing spline, but the former failed in retaining high-frequency variation in nonstationary time series and in the latter it was impossible to identify a single spline wave length that provided a good fit to both the SIE and shrub-ring data. The AR detrending procedure was performed in the dplR package (59, 60) in R where detrended series were represented by the residuals of an AR model divided by the mean of those residuals, yielding a series with white noise and a mean of 1 (60). By default in the dplR package, the order of the autoregressive model was selected automatically and independently to each series using Akaike information criterion (AIC). AR detrending was applied to both types of time series, i.e., dRCS shrub growth and raw SIE, using the common period from 1980 to 2008. In order to assure constant variance, all detrended time series were scaled (z-score) before the analyses.

### Sea Ice Data.

Pan-Arctic and regional SIE data were obtained from the National Snow and Ice Data Center, (NSIDC) (72, 73). We acquired monthly Pan-Arctic SIE data (National Oceanic and Atmospheric Administration [NOAA]/NSIDC Climate Data Record of Passive Microwave Sea Ice Concentration, Version 2; downloaded on February 24, 2017) using the following dataset link: ftp://sidads.colorado.edu/DATASETS/NOAA/G02135/. The monthly regional Sea Ice Index (Version 3) (73) was acquired from the following source: ftp://sidads.colorado.edu/DATASETS/NOAA/G02135/seaice_analysis/. Data are from the NSIDC Sea Ice Index (https://nsidc.org/data/seaice_index), obtained from the Defense Meteorological Satellite Program (DMSP) series of passive microwave remote sensing instruments. The Sea Ice Index is based on the datasets near-real-time DMSP Special Sensor Microwave/Imager (SSMI/I)-Special Sensor Microwave/Imager/Sounder (SSMIS) Daily Polar Gridded (25 × 25 km) Sea Ice Concentrations (https://nsidc.org/data/nsidc-0081) and the National Aeronautics and Space Administration-produced Sea Ice Concentrations from Nimbus-7 SMMR and DMSP SSM/I Passive Microwave Data (https://nsidc.org/data/nsidc-0051). For more information on platforms, instruments, and methods used for SIE data acquisition and processing please refer to Meier et al. (72) and the Sea Ice Index user guide (https://nsidc.org/data/g02135).

Regional SIE data were assigned to each shrub-ring location based on the shortest distance of each study site to open sea/ocean. Specifically, in our analyses, the following sea ice regions were used: Central Arctic, Hudson Bay, Baffin Sea, Beaufort Sea, East Siberian Sea, Kara Sea, Barents Sea, and Greenland Sea. A complete list of the regional SIE data assigned to each chronology is presented in *SI Appendix*, Table S2. Two gaps in monthly Pan-Arctic and regional SIE data (i.e., for December 1987 and January 1988) were filled by computing the arithmetic mean of monthly indices for adjacent years for December only (using SIE for December in years 1986 and 1988) and January only (using SIE for January in years 1987 and 1989). In order to examine more synoptic effects of sea ice extent on summer climate and shrub growth, we used seasonal SIE data instead of monthly data in both LME and SEM analyses (see below). The complete list of seasonal SIE variables used in the analyses is presented in *SI Appendix*, Table S9.

Finally, in order to obtain a more detailed understanding of the regional-scale relationships between sea ice and shrub growth, additional LME models and SEM analyses for each responder group were run using previous year first advance day of sea ice (pFAD, i.e., timing of regional sea ice freeze-up) and both previous and current year last retreat day of sea ice (pLRD and LRD, i.e., timing of regional sea ice retreat). Both measures were expressed as day of the year, thus were z-scored before the analyses for all sea ice regions. Sea ice data sources and equations for FAD and LRD for each region followed Stroeve et al. (74). Two sea ice concentration (SIC) thresholds (15% and 50%) were used for computing mean timing (i.e., day of the year) of sea ice retreat and advance in annual resolution for each region. Mean FAD and LRD values were obtained by taking an area-weighted average of grid cells that experienced both retreat and advance of sea ice for every year from 1979 to 2017. The regional division of Pan-Arctic sea ice was applied after Stroeve et al. (74) and assignment of each shrub-ring chronology to a certain sea ice region was similar as it was for regional SIE (*SI Appendix*, Table S2). LRD and FAD at the 50% SIC threshold showed greater intraseasonal variation and were better correlated with the shrub-ring data than when computed at 15% SIC. Therefore, LRD and FAD calculated at the 50% SIC threshold were retained in the final analyses. Only standardized (z-score) sea ice-related variables were used in SEM and LME analyses (see below).

### Climate Data.

Mean monthly air temperature and precipitation data were acquired from the Climatic Research Unit (CRU) time series (TS) version 4.01 using 0.5 × 0.5° grids (75). We recognize that the CRU TS dataset is not perfect and that its quality decreases with increasing latitude, particularly for precipitation (76). However, in most cases, long-term instrumental data are not available and we felt that using CRU TS data for all sites would be least likely to introduce bias into our analyses. Summer temperature and precipitation (*SI Appendix*, Table S4) were calculated using June-to-August (JJA) mean monthly CRU TS dataset. SPEI was calculated using CRU TS 4.01 temperature and precipitation data in the spei package (v1.7) (77, 78) in R. SPEI is a multiscalar drought index that is related to the balance between precipitation and potential evapotranspiration (PET) and is commonly used in tree-ring studies. In order to evaluate drought conditions across longer periods, the monthly SPEI was calculated at shorter (1 mo) and longer (12 and 24 mo) timescales and tested in SEMs. In our final models, summer SPEI represented by the arithmetic mean of monthly June-to-August SPEI calculated at a 12-mo timescale was used. PET was calculated using the Thornthwaite equation (79).

### Climate–Growth Relationships.

We began our analyses at the coarsest scale, relating Pan-Arctic SIE with shrub-ring chronologies and progressively analyzed the data at increasingly finer resolutions down to the relationships between the timing of sea ice retreat at the regional scale with the detrended ring width series of individual shrubs. We aimed to first provide a summarized understanding of shrub growth at the site-by-species level using chronologies, and to then study individual shrub responses to sea ice using linear mixed effects models. Specifically, in order to examine the potential link between sea ice and shrub growth, we first computed Pearson’s correlations between the 23 detrended (dRCS) shrub chronologies and monthly Pan-Arctic and regional SIE (*SI Appendix*, Tables S5 and S7) over the period 1979 to 2008. Additionally, we ran similar analyses using the entire chronology timespan (i.e., from year 1979 when the first SIE measurements were performed until the most recent year of each chronology) (*SI Appendix*, Table S6). The analyses were performed using monthly (from previous June to current August) and seasonal SIE data first for the entire Arctic Ocean and for the regional SIE relevant to each chronology location (*SI Appendix*, Table S2). Correlation *P* values were adjusted for multiple comparisons at the chronology level using the false discovery rate correction (80). However, shrub and chronology aggregation to each responder group (see below) was not restricted to the outcome of the correction test. Additionally, linear regression was used to assess direct relationships between each chronology and both seasonal Pan-Arctic and seasonal regional SIE (*SI Appendix*, Table S8). Based on these results, each chronology that was significantly correlated with either Pan-Arctic or regional SIE was aggregated into a specific responder group: increasers, chronologies that were negatively correlated with at least one monthly or seasonal SIE variable; and decreasers, chronologies that were positively correlated with at least one monthly or seasonal SIE variable (Fig. 1*B* and *SI Appendix*, Table S1). Chronologies that were not significantly correlated with at least one SIE variable were tagged as neutral (*SI Appendix*, Table S1) and were not included in further analysis. To statistically corroborate our grouping of shrub-ring chronologies into increasers and decreasers, we conducted a *k*-means cluster analysis, which is an unsupervised machine learning method of clustering a dataset into groups (81). This analysis was done using the correlation coefficients for each shrub-ring chronology with monthly Pan-Arctic SIE between previous June and current August (*SI Appendix*, Tables S5 and S6). The optimal number of groups was tested by minimizing within group sum of squares. This analysis confirmed the identities of the increasers and decreasers, while also revealing that the dataset supports two primary groups (*SI Appendix*, Fig. S3).

All shrub-ring chronologies, except for one (*SI Appendix*, Tables S1 and S5–S8), were significantly correlated with Arctic-wide or regional SIE. Thirteen chronologies (59% of shrubs: 201 *Betula* and 162 *Salix*) were negatively correlated with at least one monthly or seasonal SIE variable (increasers) and nine shrub chronologies (41% of shrubs: 105 *Betula* and 146 *Salix*) were positively correlated with at least one monthly or seasonal SIE variable (decreasers) (*SI Appendix*, Table S1). In order to identify very large (Pan-Arctic)-scale growth signals, all standardized shrub-ring chronologies that were assigned either to increasers or decreasers were averaged to create a mean responder-type chronology. Mean correlation (r.bar.tot) between all shrubs’ growth series per responder group was 0.094 (*n* = 363 shrubs) and 0.108 (*n* = 251 shrubs) for increasers and decreasers, with 0.971 and 0.967 EPS accordingly for the common period 1979 to 2008. Mean shrub age for increasers was 45.1 y (SD = 18.3), while the median was 44 y. Mean shrub age for decreasers was 39.4 y (SD = 15.8), with a median of 35 y. Thus, our threshold for young versus old shrubs was set at 40 y (Fig. 2 *A* and *D*). For each responder-type chronology (Fig. 1*A*), correlation coefficients over time between seasonal Pan-Arctic SIE and shrub growth were assessed using a moving correlation analyses for the common period 1980 to 2008 (Fig. 3) using the treeclim package (82) in R. These analyses allowed us to examine potential changes (both in strength and sign) of correlations between sea ice and responder-type chronologies over time. The analyses were computed using a fixed window of 20 y with 1-y moving windows. Significance of the correlation coefficients was assessed with bootstrapped resampling (1,000 iterations).

Potential differences in growth trends and climate–growth relationships (i.e., summer temperature and summer Pan-Arctic SIE) between dry vs. moist sites and *Salix* vs. *Betula* chronologies were investigated using linear regression. The differences were examined with ANOVA and slopes comparison using the “lstrends” function in the lsmeans package (83) in R. The ratio of chronologies from sites with soil moisture classified as dry versus moist was 9:4 and 4:5, respectively, for increasers and decreasers (*SI Appendix*, Table S1). Among increasers there were six *Betula* and seven *Salix* chronologies, whereas for decreasers there were three *Betula* and six *Salix* chronologies.

### Structural Equation Modeling.

In order to examine pathways by which SIE potentially affects shrub growth and to differentiate between direct and indirect effects of SIE and climate (temperature, precipitation, SPEI) on shrub growth, we used piecewise structural equation models (35, 84) (SEMs) (Fig. 6). In the first step of SEM analyses, all shrub chronologies that demonstrated a significant correlation with SIE (i.e., 22 out of 23 studied chronologies) were pooled into two groups of responders, i.e., increasers and decreasers. For each group of responders, shrub-ring chronologies at the level of site and species were used in a separate SEM analyses. Following standard dendrochronological protocol, we used chronologies rather than individual shrub-ring series in order to enhance the common signal for a given species at a given site. Thus, the final dataset consisted of 13 chronologies (i.e., 390 mean annual RWI records) for increasers and 9 chronologies (i.e., 270 mean annual RWI records) for decreasers analyzed for the period 1979 to 2008. Due to the lack of previous year sea ice data for shrub growth in 1979, all SEM analyses using sea ice data from the year preceding growth were performed for the period 1980 to 2008.

In this first step, a conceptual model was constructed in order to investigate complex (i.e., direct and indirect) relationships among SIE, climate, and shrub growth of both groups of responders. Considering regional climate and using a priori knowledge (22), we included in our SEM summer climate variables only, i.e., June-to-August temperature and precipitation. Growth relationships with dormant season climate are occasionally reported and were not included in our analyses. To investigate a possible link between declining shrub growth and moisture limitation (22, 23, 25), we included June-to-August SPEI in the model. In order to explore potential mechanisms behind divergent shrub growth responses associated with changes in sea ice and climate, the same SEM structure for each group of responders was applied. Thus, all paths, including nonsignificant ones, were retained in the final SEMs for comparative purposes.

In order to account for possible lagged effects of sea ice on climate and therefore on shrub growth, we fit separate models for seasonal SIE conditions present during the dormant season (i.e., previous autumn, winter, and spring). To further explore the connections between sea ice conditions, climate, and shrub growth, we fit models using previous year FAD (pFAD) and LRD (pLRD), as well as current year LRD. Due to relatively high pairwise correlation coefficients (i.e., >0.5 or < −0.5) among sea ice-related variables (i.e., Pan-Arctic SIE, regional SIE, pFAD, and LRD, *SI Appendix*, Figs. S10 and S11) only one sea ice variable was included in each SEM analysis and LME model. Three main potential causal pathways through which each sea ice-related variable may influence annual shrub growth were investigated in the SEM analyses, namely via 1) summer precipitation, 2) summer temperature, and 3) summer SPEI. By partitioning covariance among sea ice and climate variables, we gained insight into the pathways through which declining SIE affects tundra shrub growth.

In order to incorporate variation among the chronologies aggregated to a particular responder group, LME models for RWI, summer SPEI, temperature, and precipitation were implemented in the SEMs with a random intercept for a site included at each level of each piecewise SEM. Using this structure allowed us to test for potential site-specific variability in climate–growth and sea ice–climate relationships. We assumed linear Gaussian relationships between variables included in the model. Prior to SEM analyses, we tested each variable for normality and generated univariate density plots. To improve predictive accuracy of our SEMs, only standardized data were used. Shrub growth data (response variable) were standardized per shrub using dRCS (see *Detrending* above). Other variables were standardized per site (mean zero, unit variance) using z-scores, except for SPEI which was already represented by an index. As well, only standardized coefficient estimates are reported for piecewise SEM results (Fig. 6 and *SI Appendix*, Tables S10 and S11).

In order to specify which sea ice variable best explained the variation in shrub growth, separate SEM models were fit using seasonal regional SIE as well as regional FAD and LRD variables (*SI Appendix*, Tables S10 and S11). We were primarily interested in testing relationships between regional sea ice variables and regional climate to elucidate the indirect impact of sea ice on shrub growth. Thus, the models with the smallest direct effect of sea ice on shrub growth were the focus of the analysis for each responder group. The models for Pan-Arctic SIE were computed as well, for comparative purposes. Since those models highlighted strong direct effects of seasonal SIE on shrub growth, and actual direct effects of SIE on tundra shrub growth are unlikely, we emphasize the results of SEMs utilizing regional sea ice variables (Fig. 6 *A* and *B*). The direct path between sea ice and shrub growth in our SEMs is treated as an indication of additional indirect effects of SIE on shrub growth, i.e., unexplained variance. For instance, SIE is expected to affect numerous climate variables that may influence shrub growth, but are not available in long-term gridded climate datasets. These variables include, but are not limited to, cloud cover, fog, irradiance, wind speed, precipitation type, and the atmospheric water vapor pressure deficit. We anticipate that inclusion of these variables would reduce the significance of the direct path between SIE and shrub growth, while also increasing the explanatory power of our SEMs.

We evaluated the fit of each SEM using χ^2^, *P* value, Fisher’s *C* test, and Akaike’s information criterion corrected for small sample size (AICc) (35) (*SI Appendix*, Tables S10 and S11). The model was judged to have a good fit if *P* > 0.05, which indicates that model is consistent with the data (84). Shipley’s test of d-separation was used to investigate potentially missing paths in each piecewise SEM (35, 85). For each response variable in the SEM the level of variance explained was given using conditional R^2^ (pseudo R^2^) that takes into account both fixed and random effects (86) (Fig. 6 and *SI Appendix*, Tables S10 and S11). LME models were fit using the nlme package (87), whereas SEMs were constructed using the piecewiseSEM package (35).

### Linear Mixed Effects Models.

In addition to SEMs, the link between each sea ice variable and the growth rings of individual shrubs was verified by analyzing individual shrub-ring series hierarchically in LME models. By including random effects, LME models allowed us to account for various levels of potential nonindependence in our dataset. To account for the nested structure of shrub sampling designs across all study sites (e.g., different number of shrubs of a particular species sampled across sites) various random effects were included and tested in the LME models. Specifically, LMEs were constructed for: 1) each responder group using all shrubs (not chronologies) and all sea ice-related variables tested in SEMs as fixed effects and a random intercept for shrub nested within a site (LME1) (*SI Appendix*, Table S12); 2) each site using all shrubs and each predictor included in the SEMs as a fixed effect and year as a random effect (LME2) (*SI Appendix*, Table S13); 3) each responder group using detrended shrub growth ring series and detrended sea ice-related time series (LME3) (*SI Appendix*, Table S14), to focus on high frequency variation. LMEs for all shrubs at the responder group level used 11,239 growth rings (*n* = 363 shrubs; 201 *Betula* and 162 *Salix*) and 7,822 growth rings (*n* = 251 shrubs; 105 *Betula* and 146 *Salix*) for increasers and decreasers, respectively. LME models covered the common period of 1980 to 2008. This period was chosen in order to compare all shrubs’ growth across all sites in the most comprehensive way (i.e., for the period represented by the vast majority of shrubs included in our synthesis) (*SI Appendix*, Table S1). Having right-skewed distributions, ring width indices for each responder group were square-root transformed for final LME models (*SI Appendix*, Fig. S1 *C* and *F*). Results obtained for transformed and untransformed shrub growth data were qualitatively similar. Fixed effects were represented by the same sea ice-related variables used in the SEMs (i.e., Pan-Arctic and regional SIE, pFAD, pLRD, LRD) (*SI Appendix*, Table S9). All predictor variables were transformed to z-scores prior to the analysis (i.e., mean centered and scaled by the SD). The final random intercept structure for a responder level LME models was composed of an individual shrub nested within a site (LME1). This random intercept structure accounts for variation in means between unevenly replicated units and partitions between-site variation in shrub growth–sea ice relationships. It allowed us to assign the correct weight to each annual observation between shrub growth and sea ice among study sites and minimized the effect of (both low and high) extreme years in regional sea ice declines on relationships described. To avoid the risk of multicollinearity among predictor variables (*SI Appendix*, Figs. S10 and S11), we primarily investigated the univariate relationships between sea ice and shrub growth. In addition to the null model, we tested 11 LME models for the period 1980 to 2008 per each responder group. As our main interest was to investigate sea ice–shrub growth interactions, LME models were fit with the exact same sea ice variables used in SEM analyses. The results of LME models using all shrubs per site and species for each responder group (*SI Appendix*, Table S12) confirmed the divergent sea ice vs. shrub growth signal obtained when using chronologies in SEMs.

Additionally, individual LME models were run for each species-by-site combination separately using all shrubs, instead of a chronology at a level of a particular site (LME2) (*SI Appendix*, Table S13). A random intercept for year was included in these models. These LME models were fit using all variables used in SEMs, i.e., both sea ice and summer climate variables. The best sea ice model for each site was compared with the overall best model. The results of LME models using all shrubs per site and species confirmed the divergent sea ice vs. shrub growth signal obtained when using chronologies. Finally, LME models were run for each responder group using double detrended shrub growth series and detrended sea ice time series for all sea ice-related variables (LME3) (*SI Appendix*, Table S14 and Fig. S6 *C*, *D*, *G*, and *H*). This was done in order to account primarily for high-frequency variation in shrub and sea ice time series and to compare the interannual variability in both time series. For these models, both RWI after dRCS detrending for all shrubs and sea ice-related time series were used after AR detrending. In the final models, a random intercept for a site was included as such a structure produced a lower AIC than a nested random effects structure (i.e., shrub nested within a site).

All LMEs were fit with the R package nlme (86). Maximum likelihood was used for model fit estimations. A first-order autocorrelation structure was included in each model. Diagnostic plots were used for assessing the normality of residuals and random effects, as well as to evaluate the homogeneity of variance and the assumption of linearity. Model selection and ranking was performed using the “dredge” function in the MuMIn package (88) in R. For each LME model comparison, we ran a null model that included a random intercept only and the same random effects structure as the full models. For model selection, AICc was used, together with Akaike weights for model comparison (89) or ΔAIC (i.e., difference between best model and the corresponding null model). Additionally, the quality of LME models was quantified using marginal and conditional pseudo R^2^. These model statistics were computed for each model using the “r.squaredGLMM” function in the MuMIn package (88) in R, taking into consideration possible constraints of these statistics (86).

## Supplementary Material

Supplementary File

Supplementary File

## Data Availability

All individual shrub ring data used in this manuscript are publicly available from the Dryad Digital Repository, https://doi.org/10.5061/dryad.kh1893248. Example code used for the analyses is available from Dataset S1.
