# Supplementary material for: Divergence of Arctic shrub growth associated with sea ice decline
Source: Proc Natl Acad Sci U S A. 2020 Dec 14;117(52):33334–44. doi: 10.1073/pnas.2013311117 (PMC7776857; doi:10.1073/pnas.2013311117)
Supplement: Supplementary File [file pnas.2013311117.sapp.pdf]

PNAS
www.pnas.org

Supplementary Information for:

Divergence of Arctic shrub growth associated with sea ice decline

Agata Buchwal[a,b,1], Patrick F. Sullivan[c], Marc Macias-Fauria[d], Eric Post[e], Isla Myers-Smith[f], Julienne C. Stroeve[g], Daan Blok[h], Ken D. Tape[i], Bruce C. Forbes[j], Pascale Ropars[k,l], Esther Lévesque[m], Bo Elberling[n], Sandra Angers-Blondin[f], Joseph S. Boyle[f], Stéphane Boudreau[o], Noémie Boulanger-Lapointe[p], Cassandra Gamm[c], Martin Hallinger[q], Grzegorz Rachlewicz[a,r], Amanda Young[s,t], Pentti Zetterberg[u], and Jeffrey M. Welker[v,b]

[a]Institute of Geoecology and Geoinformation, Adam Mickiewicz University, B. Krygowskiego 10, 61-680 Poznan, Poland; [b]Department of Biological Sciences, University of Alaska Anchorage, 3151 Alumni Loop, 99508 Anchorage, Alaska, USA; [c]Environment and Natural Resources Institute, University of Alaska Anchorage, Anchorage, AK, USA; [d]School of Geography and the Environment, University of Oxford, OX1 3QY Oxford, UK; [e]Department of Wildlife, Fish, and Conservation Biology, University of California, Davis, 95616 USA; [f]School of GeoSciences, University of Edinburgh, EH9 3FF Edinburgh, UK; [g]University of Manitoba, Winnipeg and the National Snow and Ice Data Center, Cooperative Institute for Research in Environmental Sciences, University of Colorado at Boulder, Boulder, Colorado, USA; [h]Dutch Research Council (NWO), The Hague, 93460, The Netherlands; [i]Geophysical Institute, 2156 N Koyukuk Drive, University of Alaska Fairbanks, 99775 Fairbanks, Alaska, USA; [j]Arctic Centre, University of Lapland, Box 122, FI-96101 Rovaniemi, Finland; [k]Chaire de recherche du Canada en biodiversité nordique, Université du Québec à Rimouski, QC, G5L 3A1, Canada; [l]Centre for Indigenous Peoples' Nutrition and Environment, McGill University, QC, H9X 3V9, Canada; [m]Département des Sciences de l'environnement et Centre d'études nordiques, Université du Québec à Trois-Rivières, QC, G8Z 4M3, Canada; [n]Center for Permafrost (CENPERM), Department of Geoscience and Natural Resource Management, University of Copenhagen, Copenhagen, Øster Voldgade 10, DK-1350 Copenhagen, Denmark; [o]Département de biologie et Centre d'études nordiques, Université Laval, 1045 Avenue de la Médecine, G1V 0A6 Québec, Canada; [p]Department of Geography, University of British Columbia, 1984 West Mall, V6T 1Z2 Vancouver, BC, Canada; [q]Biology Department, Swedish Agricultural University (SLU), SE-750 07 Uppsala, Sweden; [r]Department of Health and Environmental Science, Xi'an Jiaotong-Liverpool University, Ren'ai Road 111, 215123 Suzhou, China; [s]Toolik Field Station, Institute of Arctic Biology, University of Alaska Fairbanks, USA; [t]Department of Geography, Pennsylvania State University, USA; [u]Laboratory of Dendrochronology, School of Forest Sciences, University of Eastern Finland, Joensuu, Finland; [v]Department of Ecology & Genetics, University of Oulu and UArctic, Oulu, Finland

Corresponding author: Agata Buchwal
Email: kamzik@amu.edu.pl

This PDF file includes:

Figures S1 to S11

www.pnas.org/cgi/doi/10.1073/pnas.2013311117

Tables S1 to S14
SI References

Other supplementary materials for this manuscript include the following:

All individual shrub ring data are available from the Dryad Digital Repository:
https://doi.org/10.5061/dryad.kh1893248
(Buchwal et al. 2020), Deposited date: November 24, 2020.

Buchwal, Agata et al. (2020), Data from: Divergence of Arctic shrub growth associated with sea ice decline, Dryad, Dataset, https://doi.org/10.5061/dryad.kh1893248

Datasets S1 (Example R code used for the analyses)

Supplementary Figures

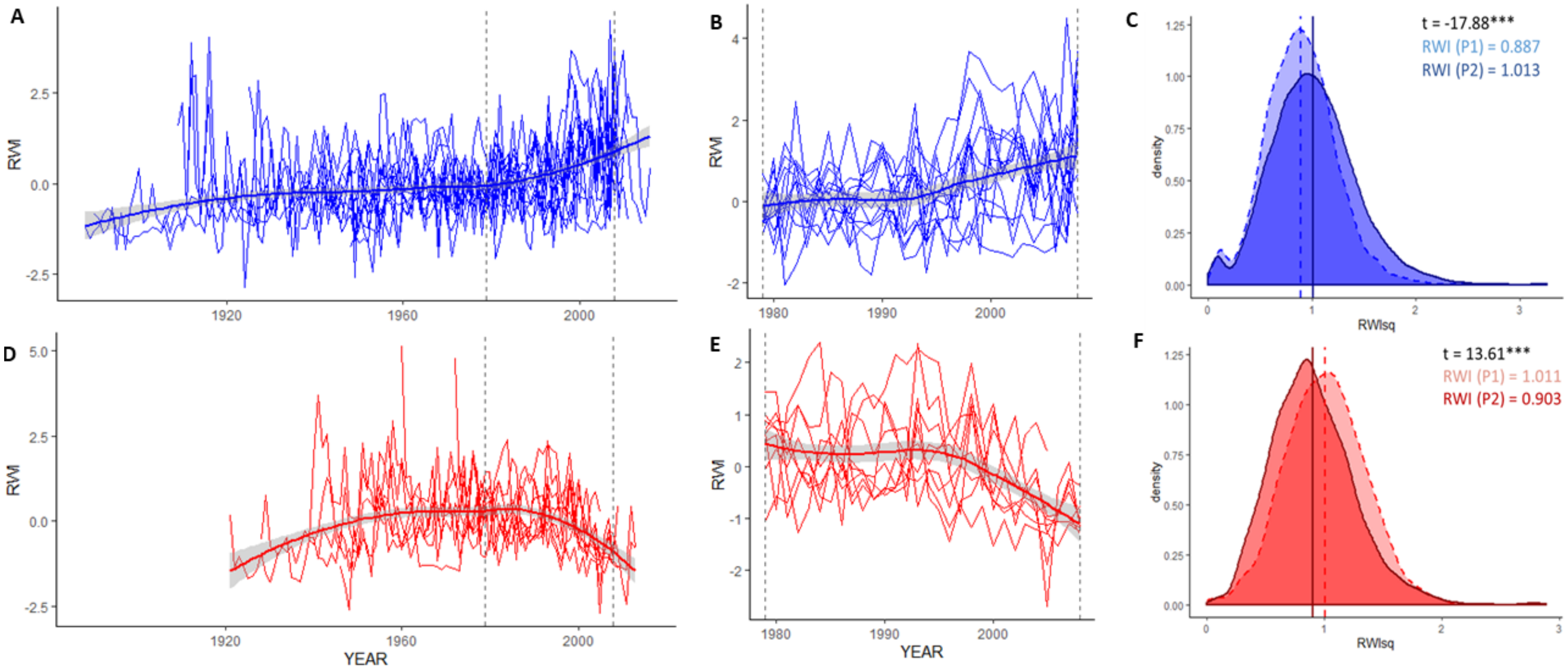

Fig. S1. Overview of *increasers* (A, B) and *decreasers* (D, E) standardized (dRCS) shrub chronologies analyzed in the synthesis together with changes in mean RWI for all *increasers* (C) and *decreasers* (F) shrubs. Overview of entire chronologies timespan (**A**, **C**), together with study period 1979-2008 (**B**, **E**) indicated by vertical dashed lines; *increasers* (blue lines) = 13 chronologies (363 shrubs); *decreasers* (red lines) = 9 chronologies (251 shrubs). Smoothed conditional means with 95% confidence intervals are marked for each responder group. Mean correlation between chronologies (1979-2008) equaled 0.108 and 0.4 for *increasers* and *decreasers*, respectively. Changes in mean RWI (**C**, **F**) between early (P1=1980-1993; dashed lines and lighter colors) and recent (P2=1994-2008; solid lines and darker colors) period for all shrubs analyzed for (**C**) *increasers* (n=11,239 annual growth records, with 43% and 57% shrubs assigned to early and recent period respectively) and (**F**) *decreasers* (n=7,822 annual growth records, with 42% and 58% shrubs assigned to early and recent period respectively). RWIsq - ring-width index after transformation (square-root). Results of Welch two sample t-test, together with mean RWI for each period, are presented for each responder group in the top right. *** *P*<0.001

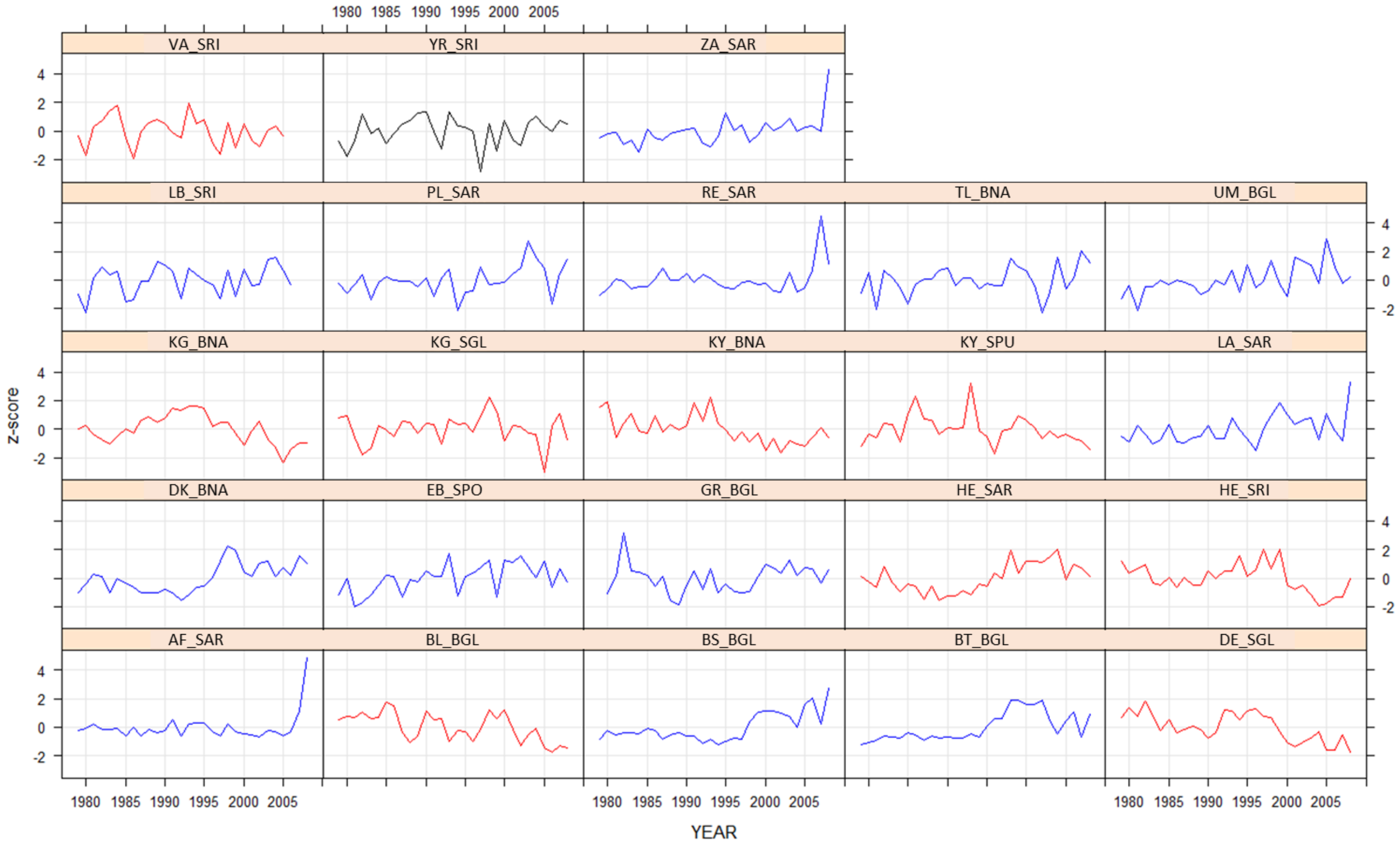

Fig. S2. Overview of 23 raw shrub ring chronologies (scaled for comparison) used in SIE-shrub growth relationship analyses for common period (1979-2008). Blue curves - *increasers*; red curves - *decreasers*; black curve - *neutral* chronology. For full site names and descriptive statistics for each chronology see Table S1

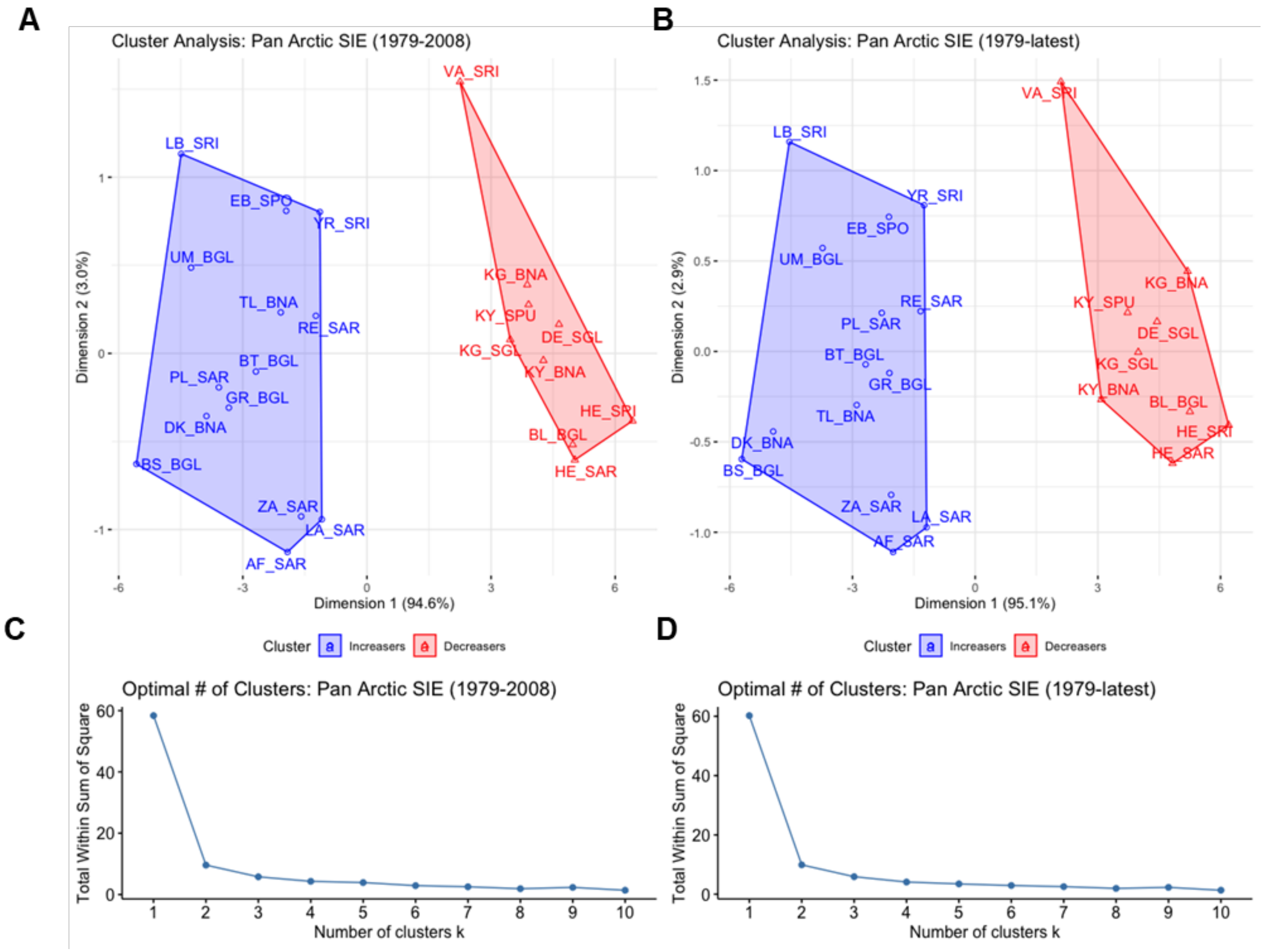

Fig. S3. Principal components analysis of the results of a *k*-means cluster analysis designed to group shrub radial growth responses to variation in monthly Pan-Arctic sea ice extent (SIE, 23 shrub-ring chronologies, dRCS detrending). Correlations coefficients for each chronology were obtained using monthly Pan-Arctic SIE (from previous June to current August) for (a, c) common period (1979-2008; Table S5) and (b, d) entire chronology timespan (Table S6). Blue color - *increasers*; red color - *decreasers*. Analysis of the optimal number of groups (c, d) showed strong support for just two major groups of responders. Thus, the YR chronology, which was identified as a *neutral* responder, because it was not significantly correlated with SIE during any months, was identified as a marginal increaser in this analysis. The YR chronology was excluded from subsequent analyses. For full chronology names see Table S1.

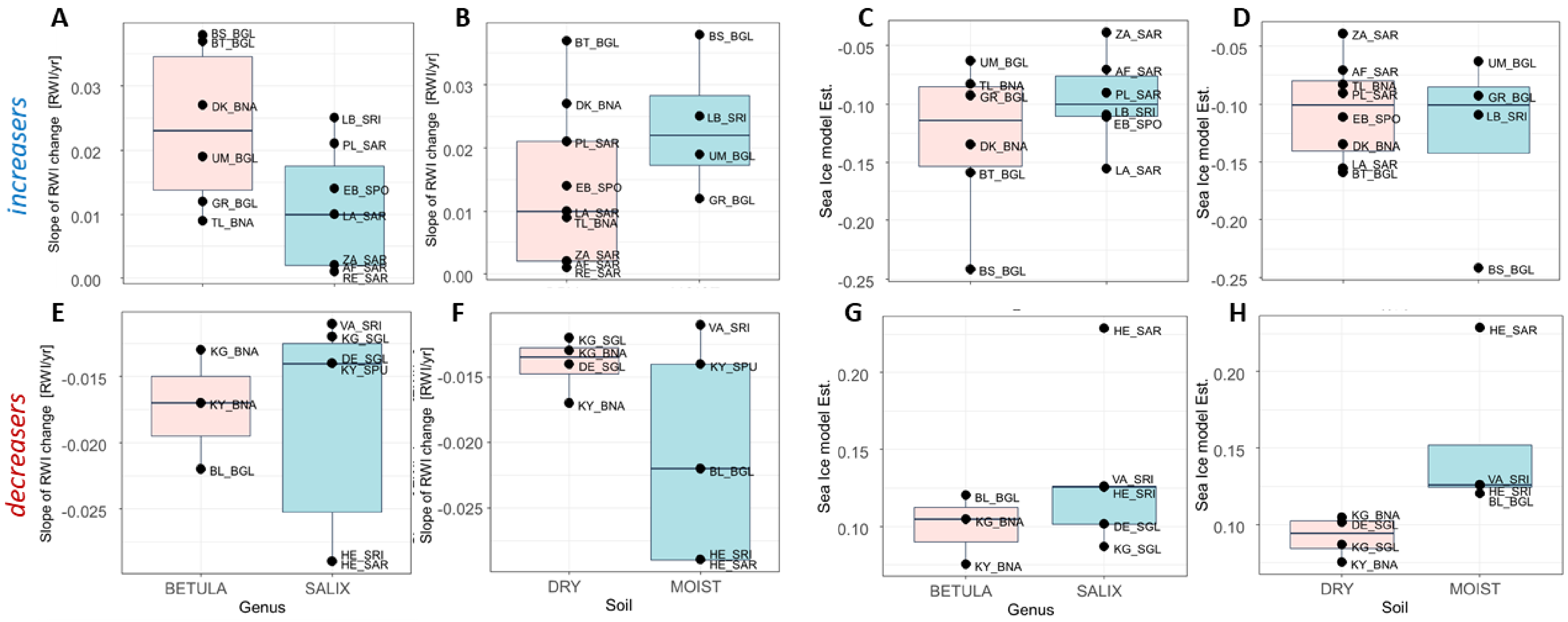

Fig. S4. Variation in mean standardized radial growth (RWI - ring width index, 1979-2008) changes together with variation in coefficient estimates for the best sea ice model (1980-2008) obtained for *increasers* (A-D) and *decreasers* (E-H) chronologies. Results are shown with distinction between Betula and Salix chronologies (**A, C, E, G**), and dry and moist soil types (**B, D, F, H**). Model estimates for the best sea ice model were obtained from linear mixed effect models using all shrubs (instead of chronologies, Table S13) with a random intercept for a year. See Table S1 for full chronology names.

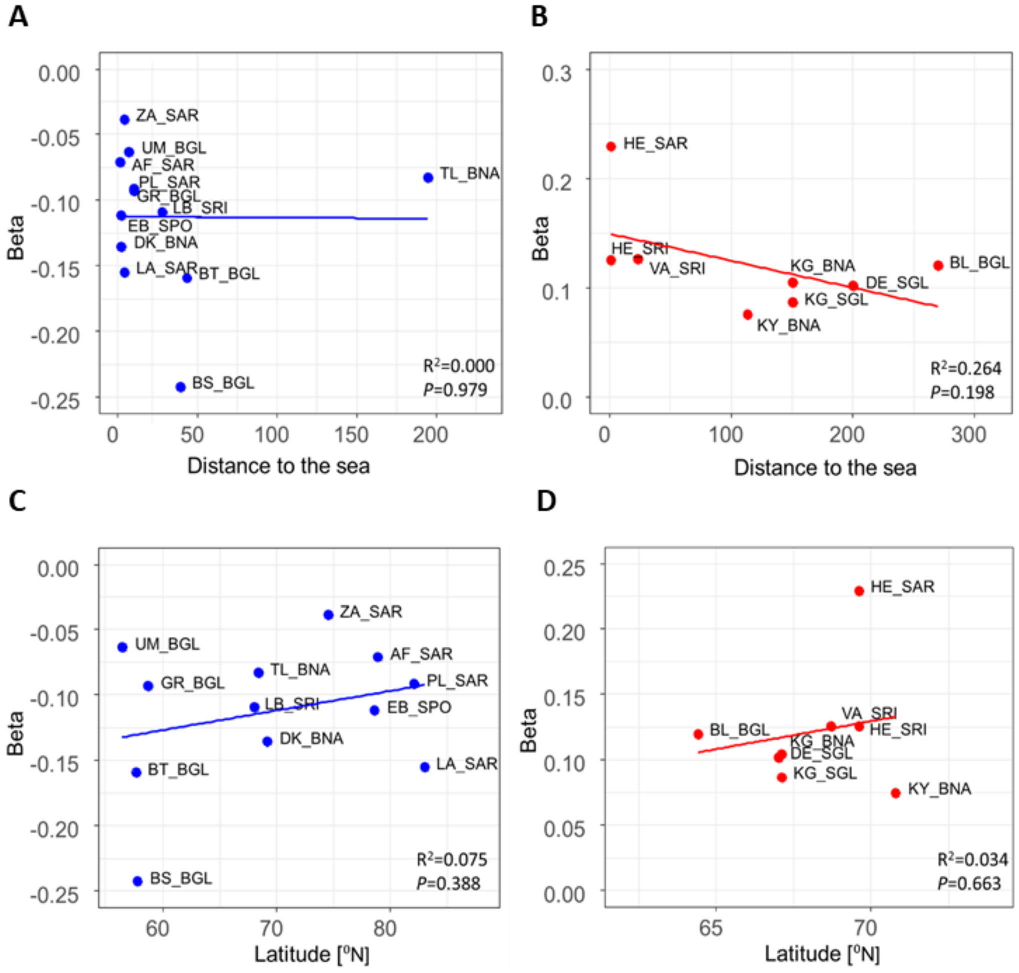

Fig. S5. Variation in coefficient estimates for the best sea ice model (Beta) obtained for each *increaser* (A, C) and *decreaser* (B, D) chronology (1979-2008) in relation to distance from the open sea (A, B) and latitude (C, D). Model estimates were obtained from linear mixed effect models using all shrubs (instead of chronologies, Table S13) with a random intercept for a year. See Table S1 for full chronology names.

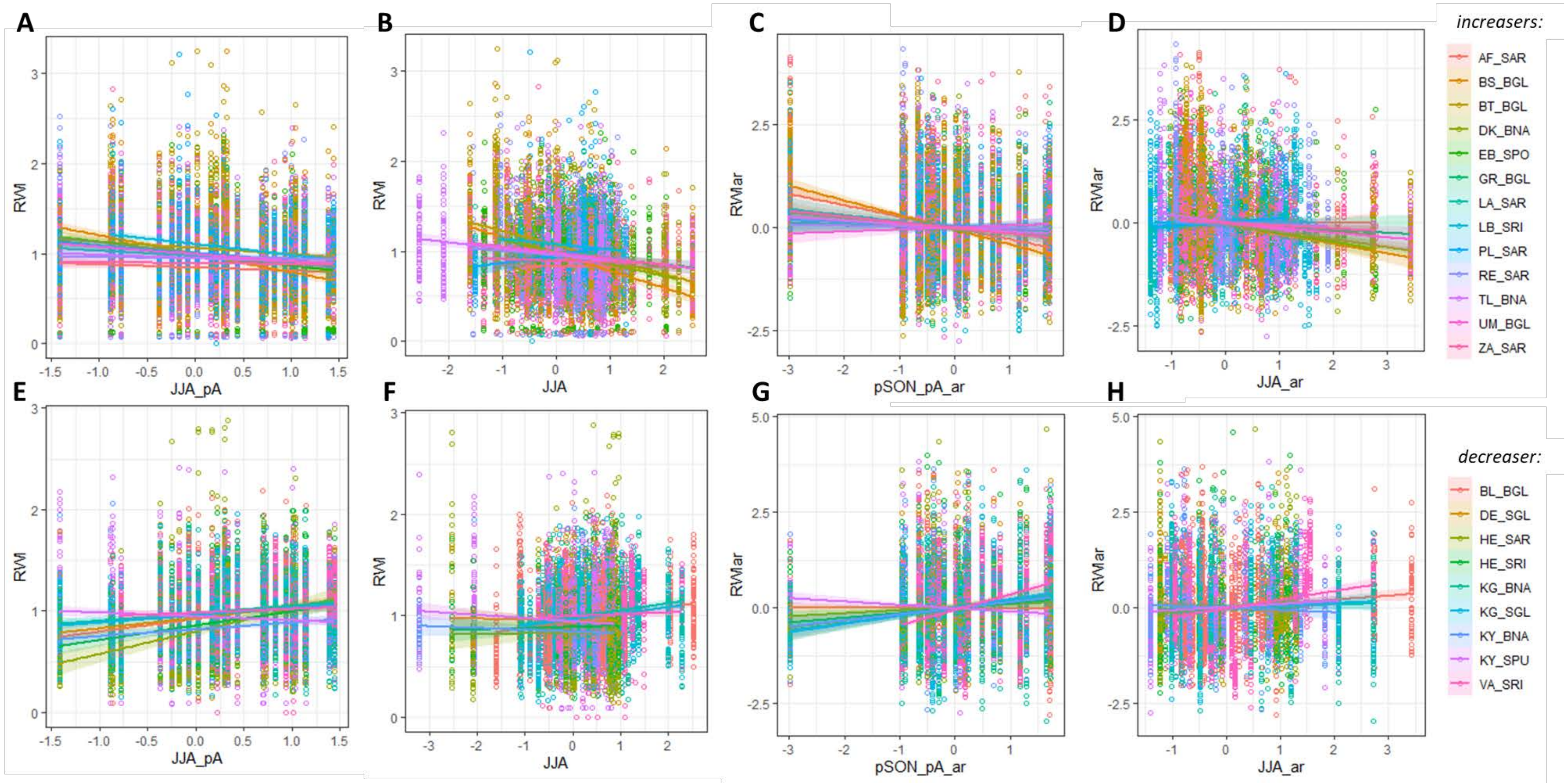

Fig. S6. Relationships between *increasers* (A-D) and *decreasers* (E-H) all shrubs annual radial growth (1980-2008, grouped by chronology) and selected sea ice variables: (**A, E**) mean Pan-Arctic sea ice extent (SIE) for June to August (JJA) period; (**B, F**) mean regional JJA SIE; (**C, G**) mean previous autumn SIE detrended; (**D, H**) mean regional JJA SIE detrended. RWI - ring width indices after square root transformation and diameter RCS detrending; RWIar - ring width indices after AR detrending. All predictors were standardized (z-score). For full model statistics (at the responder group level) please refer to Table S12 (raw sea ice data) and Table S14 (AR detrended shrub and sea ice data).

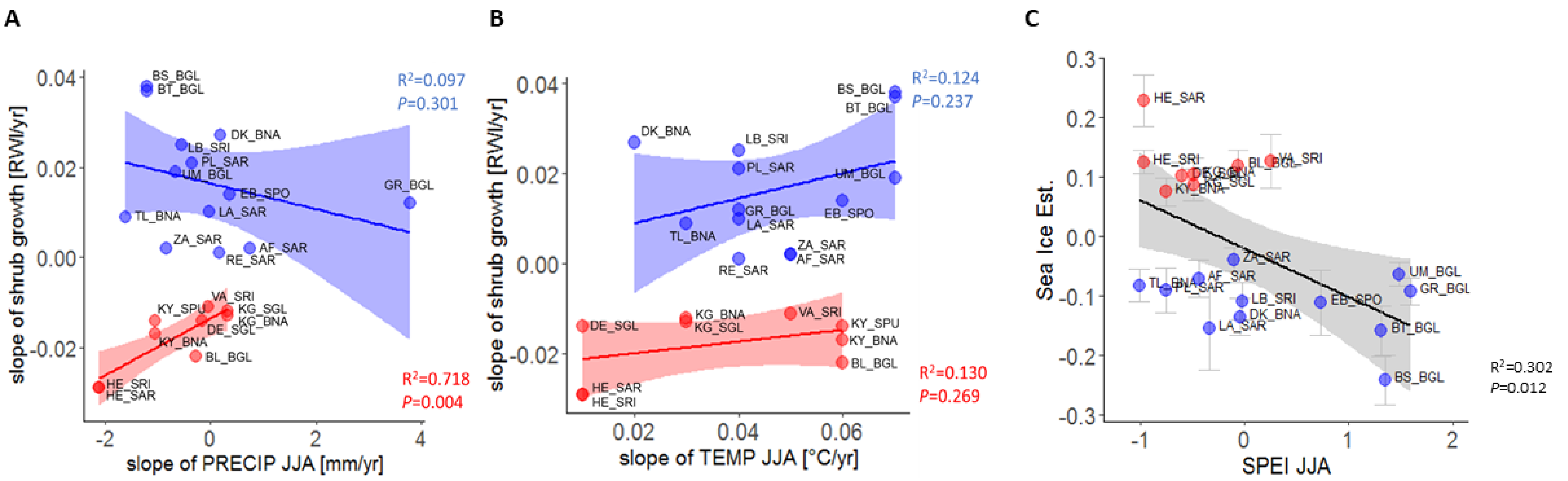

Fig. S7. (A) Relationship between slopes of shrub radial growth and slopes of June to August precipitation, and (B) June to August temperature for each *increaser* (blue points) and *decreaser* (red points) chronology (1979-2008). (C) Relationship between coefficient estimates (with associated standard errors) for the best sea ice model (Sea Ice Est.) and mean June to August SPEI obtained for each *increaser* (blue points) and *decreaser* (red points) chronology (1979-2008). Sea Ice model estimates at a site level were obtained from linear mixed effect models using all shrubs (instead of chronologies) with a random intercept for a year (Table S13).

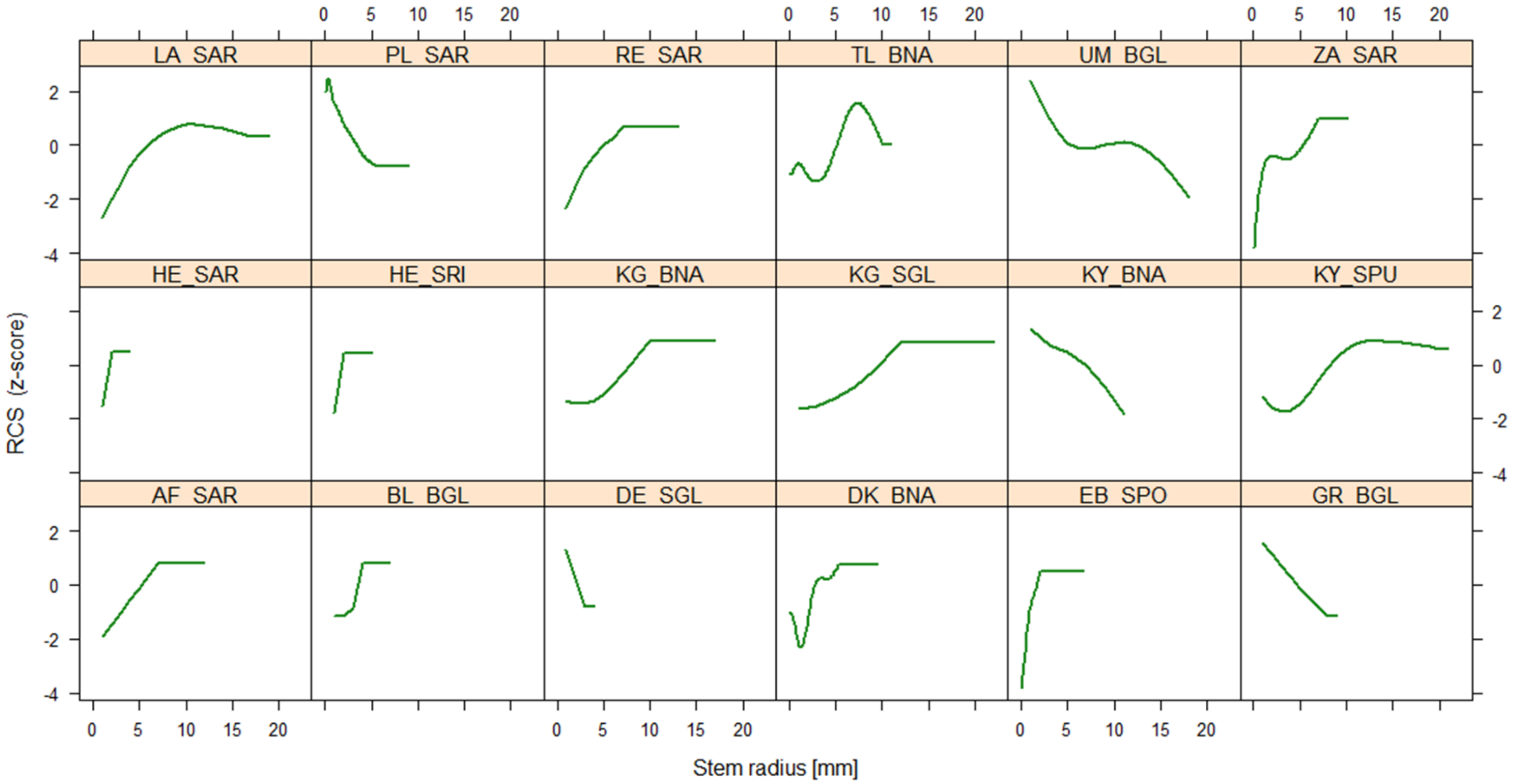

Fig. S8. Relationship between mean ring-widths and shrub stem radius. Relationship between mean ring-widths (Y axis, scaled for comparison) and shrub stem radius (X axis) for shrub ring chronologies used the synthesis with the stem radius up to 20 mm. Relationship is presented using smoothed detrending curve (RCS, diameter based) calculated for each chronology using all shrubs. For full site names see Table S1.

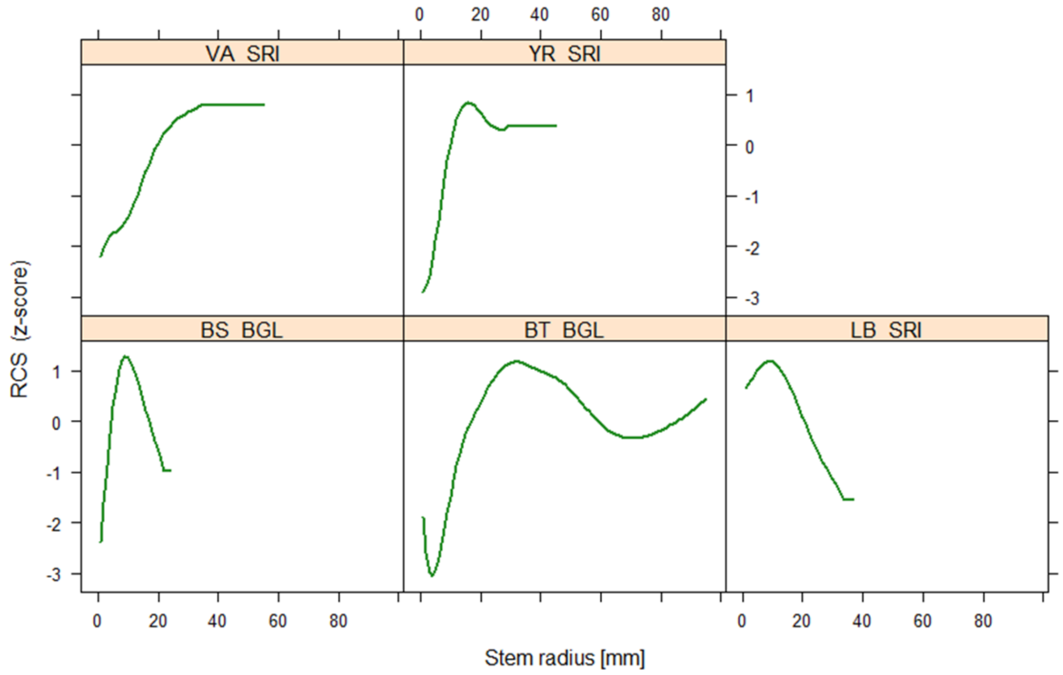

Fig. S9. Relationship between mean ring-widths and shrub stem radius. Relationship between mean ring-widths (Y axis, scaled for comparison) and shrub stem radius (X axis) for shrub ring chronologies used the synthesis with the stem radius greater than 20 mm. Relationship is presented using smoothed detrending curve (RCS, diameter based) calculated for each chronology using all shrubs. For full site names see Table S1.

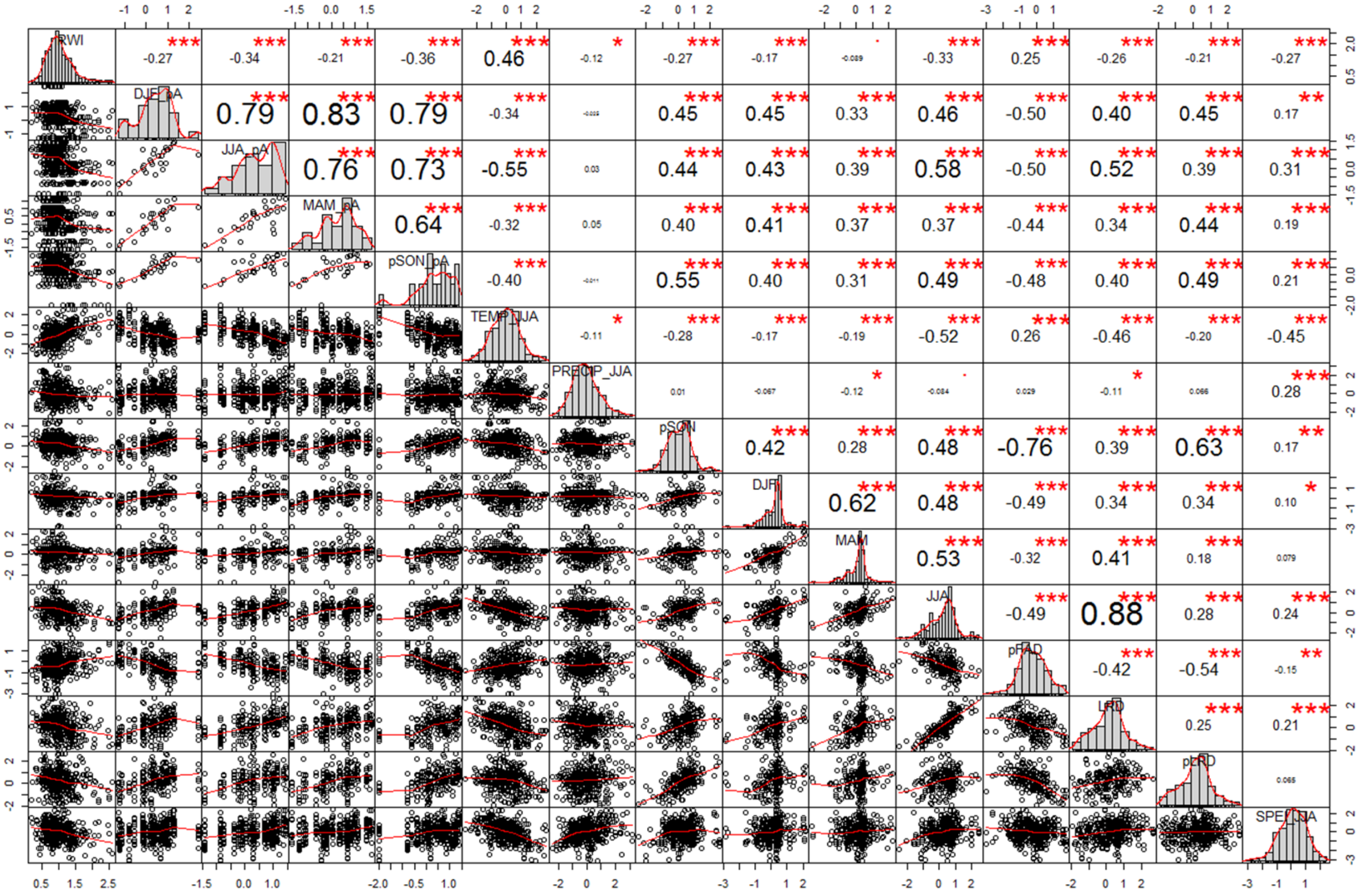

Fig. S10. Correlation matrix for response variable (RWI, i.e., mean annual ring width index for 13 *increaser's* chronologies, n=390 annual records, period 1979-2008) and predictor variables used in SEM. All predictors were standardized using z-score. For full name of predictors see Table S9. On the upper right corner Pearson's correlation coefficients between all variables are visualized with the significance level denoted with asterisks (i.e., **p*<0.05, ** *p*<0.01, *** *p*<0.001). On the bottom left corner bivariate scatterplots with fitted lines are presented.

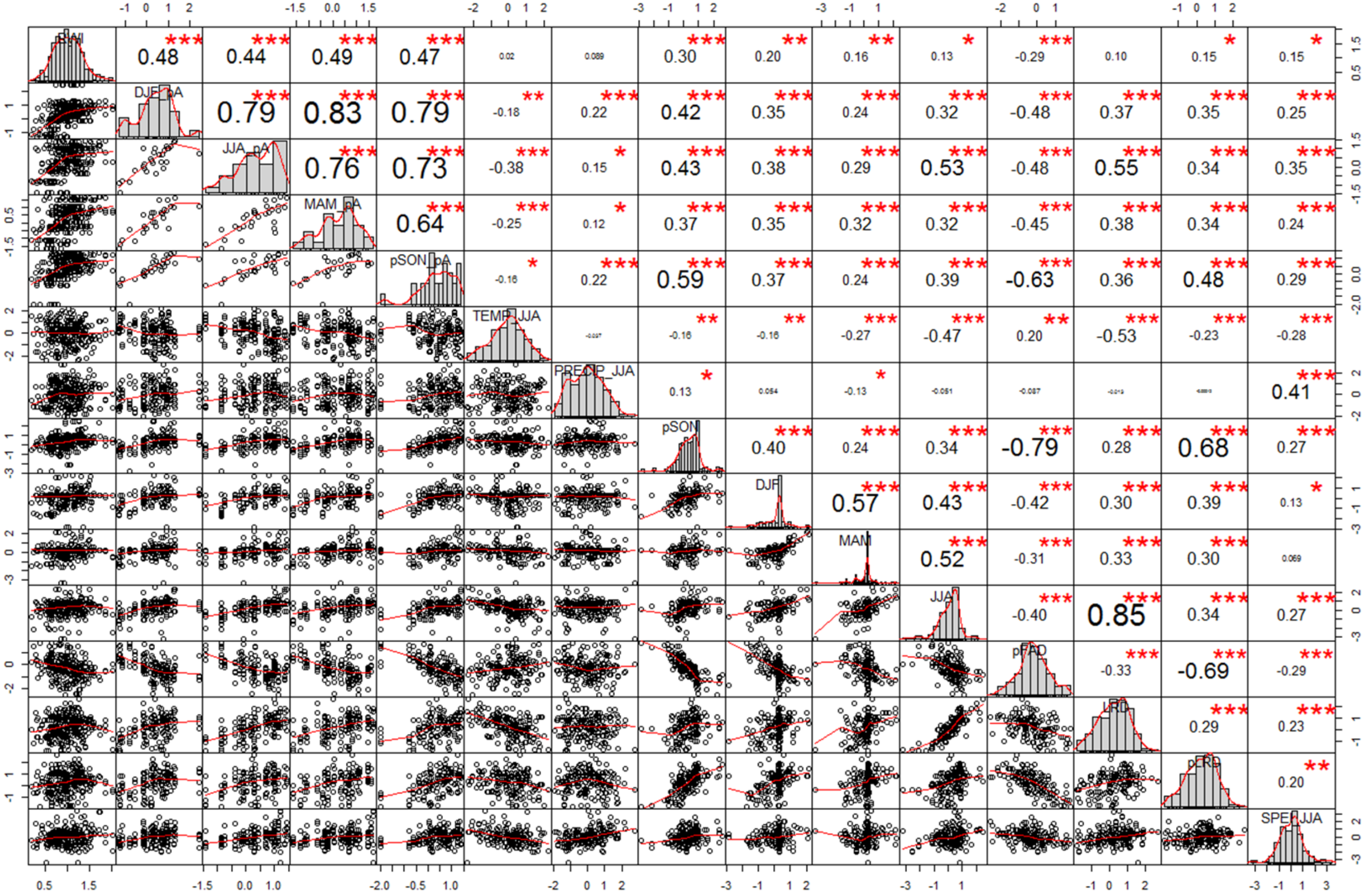

Fig. S11. Correlation matrix for response variable (RWI, i.e., mean annual ring width index for nine *decreaser's* chronologies, n=270 annual records, period 1979-2008) and predictor variables used in SEM. All predictors were standardized using z-score. For full name of predictors see Table S9. On the upper right corner Pearson's correlation coefficients between all variables are visualized with the significance level denoted with asterisks (i.e., * p<0.05, ** p<0.01, *** p<0.001). On the bottom left corner bivariate scatterplots with fitted lines are presented.

Supplementary Tables

Table S1. Characteristics of shrub chronologies included in the synthesis with responder type indication (blue font - *increaser*; red font - *decreaser*; black bold font - *neutral*). YR site, as a neutral responder, was not included in the final analyses. Types of soil moisture classes (D - dry; M - moist, W - wet) for each site were assessed after Myers-Smith et al. (2015) (3), with moist and wet classes pooled into moist soil type. Sampling design after Sullivan & Csank (2016) (11): SEL- selective; DOM - dominant; STR - stratified; RAN - random. Shrubs' sectioning method types: SS - serial sectioning; S - stem only; LS - largest stem; RC - root collar only. Descriptive statistics are presented for the study periods (1979-2008). MRW - mean ring width; sd - standard deviation; MS - mean sensitivity; AR(1) - first-order autocorrelation; rbar.tot - mean pairwise correlation between all cross-sections; EPS - expressed population signal.

Chronology ID	Site name/ Species	Latitude (N)	Longitude (E/-W)	Soil moisture class	Sampling design	Shrubs' sectioning method	# shrubs	# cross-sections	first YR	last YR	MRW [mm]	sd [mm]	MS	AR(1)	raw CRN rbar.tot	raw CRN EPS	standardized CRN rbar.tot	standardized CRN EPS	Data contributors	Reference
**AF_SAR**	Alexandra Fiord/ *Salix arctica*	78.9	-75.9	D	RAN	RC	29	29	1937	2008	0.09	0.063	0.719	0.102	0.118	0.795	0.116	0.792	Boulanger-Lapointe N.	(1)
**BL_BGL**	Baker Lake/ *Betula glandulosa*	64.4	-95.9	M	RAN	LS	44	77	1946	2010	0.115	0.062	0.382	0.563	0.313	0.969	0.328	0.971	Lévesque E.	in prep.
**BS_BGL**	Boniface River/ *Betula glandulosa*	57.7	-76.1	M	DOM	RC	39	39	1949	2009	0.659	0.462	0.471	0.541	0.309	0.935	0.326	0.939	Ropars P., Boudreau S.	(2)
**BT_BGL**	Boniface River / *Betula glandulosa*	57.7	-76.1	D	DOM	RC	76	76	1915	2009	0.572	0.371	0.454	0.574	0.548	0.988	0.488	0.985	Ropars P., Boudreau S.	(2)
**DE_SGL**	Dempster/ *Salix glauca*	67.0	-136.2	D	DOM, SEL, STR	S	15	15	1972	2008	0.052	0.035	0.401	0.451	0.475	0.908	0.32	0.837	Myers-Smith I.	(3)
**DK_BNA**	Disko Island/ *Betula nana*	69.2	-53.3	D	RAN, DOM	SS	15	31	1888	2011	0.095	0.057	0.472	0.572	0.427	0.915	0.424	0.913	Buchwal A., Rachlewicz G., Elberling B.	(4)
**EB_SPO**	Ebbadalen/ *Salix polaris*	78.6	16.5	D	RAN	SS	10	25	1941	2010	0.062	0.04	0.781	0.108	0.34	0.855	0.305	0.83	Buchwal A., Rachlewicz G.	(5)
**GR_BGL**	George River/ *Betula glandulosa*	58.7	-65.9	M	RAN	LS	17	22	1980	2011	0.301	0.107	0.304	0.316	0.228	0.807	0.198	0.777	Lévesque E.	in prep.
**HE_SAR**	Herschel Island/ *Salix arctica*	69.6	-138.9	M	DOM, SEL, STR	S, RC	24	24	1954	2008	0.068	0.032	0.384	0.339	0.228	0.825	0.259	0.849	Myers-Smith I., Angers-Blondin S.	(3)
**HE_SRI**	Herschel Island/ *Salix richardsonii*	69.6	-138.9	M	DOM, SEL, STR	S, RC	17	17	1960	2008	0.051	0.025	0.359	0.349	0.036	0.355	0.249	0.829	Myers-Smith I., Angers-Blondin S.	(3)
**KG_BNA**	Kangerlussuaq/ *Betula nana*	67.1	-50.3	D	RAN, DOM	RC	42	42	1903	2013	0.178	0.081	0.446	0.368	0.181	0.892	0.239	0.921	Sullivan P., Post E., Gamm C., Young A.	(6)
**KG_SGL**	Kangerlussuaq/ *Salix glauca*	67.1	-50.3	D	RAN, DOM	RC	32	32	1935	2013	0.291	0.126	0.402	0.372	0.185	0.854	0.246	0.894	Sullivan P., Post E., Gamm C., Young A.	(6)
**KY_SPU**	Kytalyk Reserve/ *Salix pulchra*	70.8	147.5	M	RAN, DOM	SS	19	19	1928	2009	0.087	0.056	0.464	0.421	0.234	0.849	0.2	0.821	Blok D.	(7)
**KY_BNA**	Kytalyk Reserve/ *Betula nana*	70.8	147.5	D	RAN, DOM	RC	19	19	1939	2008	0.182	0.112	0.479	0.416	0.345	0.909	0.377	0.92	Blok D.	(7)
**LA_SAR**	Lake A/ *Salix arctica*	83.0	-75.4	D	RAN	RC	19	19	1890	2008	0.178	0.118	0.739	0.099	0.401	0.926	0.366	0.915	Boulanger-Lapointe N.	(1)
**LB_SRI**	Laborovaya/ *Salix lanata*	68.0	68.0	M/W	RAN, DOM	RC	29	29	1934	2006	0.636	0.257	0.398	0.386	0.262	0.909	0.357	0.94	Forbes B. C, Macias-Fauria M., Zetterberg P.	(8)
**PL_SAR**	Peary Land/ *Salix arctica*	82.1	-33.0	D	RAN, DOM	S, SS	10	24	1925	2014	0.153	0.078	0.542	0.184	0.249	0.777	0.241	0.773	Buchwal A., Elberling B.	(9)
**RE_SAR**	Resolute/ *Salix arctica*	74.7	-94.9	D	RAN	RC	31	31	1948	2008	0.187	0.15	0.807	0.114	0.097	0.758	0.108	0.779	Boulanger-Lapointe N.	(1)
**TL_BNA**	Toolik Lake/ *Betula nana*	68.4	-149.9	D	RAN, DOM	SS	29	58	1905	2016	0.132	0.081	0.578	0.322	0.191	0.872	0.201	0.879	Buchwal A., Welker J.	in prep.
**UM_BGL**	Umiujuaq/ *Betula glandulosa*	56.5	-76.4	M	RAN	LS	25	25	1950	2010	0.339	0.154	0.44	0.378	0.216	0.855	0.219	0.858	Lévesque E.	in prep.
**VA_SRI**	Varandei/ *Salix lanata*	68.7	58.5	M	RAN, DOM	RC	39	39	1921	2005	0.895	0.417	0.536	0.285	0.546	0.979	0.57	0.981	Forbes B. C, Macias-Fauria M., Zetterberg P.	(10)
**YR_SRI**	Yuribei River/ *Salix lanata*	68.9	70.2	W	RAN, DOM	RC	27	27	1911	2009	0.619	0.267	0.437	0.306	0.35	0.935	0.344	0.934	Forbes B. C, Macias-Fauria M., Zetterberg P.	(8)
**ZA_SAR**	Zackenberg/ *Salix arctica*	74.5	-20.5	D	RAN	RC	34	34	1909	2010	0.108	0.077	0.686	0.062	0.091	0.751	0.101	0.772	Boulanger-Lapointe N.	(1)
						**min**	10	15	1888	2005	0.051	0.025	0.304	0.062	0.036	0.355	0.101	0.772		
						**max**	76	77	1980	2016	0.895	0.462	0.807	0.574	0.548	0.988	0.57	0.985		
						**mean**	28	33	1932	2010	0.263	0.14	0.508	0.332	0.277	0.853	0.286	0.874		
						**sum**	641	753												

Table S2. List of shrub chronologies included in the synthesis together with the geographical coordinates and regional sea ice province assigned. Mean day of the year (DOY) for the first advanced day (FAD) and the last retreat day (LRD) together with 95% confidence intervals was calculated after Stroeve et al. (2016) (12).

Chronology ID	Responder	Site name	Species	Latitude (N)	Longitude (E/-W)	REGIONAL SEA ICE province used:	mean DOY of FAD (95% CI)	mean DOY of LRD (95% CI)
AF_SAR	*increaser*	Alexandra Fiord	*Salix arctica*	78.9	-75.9	Baffin Sea	317 (314-321)	182 (179-185)
BL_BGL	*decreaser*	Baker Lake	*Betula glandulosa*	64.4	-95.9	Hudson Bay	335 (332-338)	176 (173-179)
BS_BGL	*increaser*	Boniface River research station	*Betula glandulosa*	57.8	-76.2	Hudson Bay	335 (332-338)	176 (173-179)
BT_BGL	*increaser*	Boniface River research station	*Betula glandulosa*	57.7	-76.1	Hudson Bay	335 (332-338)	176 (173-179)
DE_SGL	*decreaser*	Dempster	*Salix glauca*	67.0	-136.2	Beaufort Sea	293 (289-298)	189 (182-195)
DK_BNA	*increaser*	Disko Island	*Betula nana*	69.2	-53.3	Baffin Sea	317 (314-321)	182 (179-185)
EB_SPO	*increaser*	Ebbadalen	*Salix polaris*	78.6	16.5	Greenland Sea	324 (318-331)	181 (177-186)
GR_BGL	*increaser*	Kangiqsualujjuaq (George River)	*Betula glandulosa*	58.7	-65.9	Hudson Bay	335 (332-338)	176 (173-179)
HE_SRI	*decreaser*	Herschel Island	*Salix richardsonii*	69.6	-138.9	Beaufort Sea	293 (289-298)	189 (182-195)
HE_SAR	*decreaser*	Herschel Island	*Salix arctica*	69.6	-138.9	Beaufort Sea	293 (289-298)	189 (182-195)
KG_BNA	*decreaser*	Kangerlussuaq	*Betula nana*	67.1	-50.3	Baffin Sea	317 (314-321)	182 (179-185)
KG_SGL	*decreaser*	Kangerlussuaq	*Salix glauca*	67.1	-50.3	Baffin Sea	317 (314-321)	182 (179-185)
KY_SPU	*decreaser*	Kytalyk Reserve	Salix pulchra	70.8	147.5	East Siberian	286 (282-291)	201 (197-206)
KY_BNA	*decreaser*	Kytalyk Reserve	*Betula nana*	70.8	147.5	East Siberian	286 (282-291)	201 (197-206)
LA_SAR	*increaser*	Lake A	*Salix arctica*	83.0	-75.4	Central Arctic	334 (317-352)	206 (190-223)
LB_SRI	*increaser*	Laborovaya	*Salix lanata*	68.0	68.0	Kara Sea	313 (308-318)	189 (185-194)
PL_SAR	*increaser*	Peary Land	*Salix arctica*	82.1	-33.0	Central Arctic	334 (317-352)	206 (190-223)
RE_SAR	*increaser*	Resolute	*Salix arctica*	74.7	-94.9	Canadian Archipelago	293 (291-296)	192 (189-196)
TL_BNA	*increaser*	Toolik Lake	*Betula nana*	68.4	-149.9	Beaufort Sea	293 (289-298)	189 (182-195)
UM_BGL	*increaser*	Umiujuaq	*Betula glandulosa*	56.5	-76.4	Hudson Bay	335 (332-338)	176 (173-179)
VA_SRI	*decreaser*	Varandei	*Salix lanata*	68.7	58.5	Barents Sea	342 (335-348)	159 (153-164)
ZA_SAR	*increaser*	Zackenberg	*Salix arctica*	74.5	-20.5	Greenland Sea	324 (318-331)	181 (177-186)

Table S3. Mean standardized ring width index (RWI) for early (1979-1993) and recent (1994-2008) period for each chronology. Δ RWI represents difference between early and recent period. P-value from the Welch two sample t-test is reported. Values marked in bold indicate a statistically significant difference (*p*<0.05).

Responder	Chronology ID	Site name	Species	mean RWI (1979-1993)	mean RWI (1994-2008)	Δ RWI	*P*-value
increaser	AF_SAR	Alexandra Fiord	*Salix arctica*	0.80	0.79	0.00	0.967
increaser	BS_BGL	Boniface River	*Betula glandulosa*	0.74	1.29	**0.55**	**0.004**
increaser	BT_BGL	Boniface River	*Betula glandulosa*	0.81	1.60	**0.79**	**0.000**
increaser	DK_BNA	Disko Island	*Betula nana*	0.73	1.33	**0.60**	**0.000**
increaser	EB_SPO	Ebbadalen	*Salix polaris*	0.97	1.11	0.14	0.322
increaser	GR_BGL	Kangiqsualujjuaq (George River)	*Betula glandulosa*	0.91	1.04	0.13	0.165
increaser	LA_SAR	Lake A	*Salix arctica*	0.99	1.11	0.13	0.434
increaser	LB_SRI	Laborovaya	*Salix lanata*	1.04	1.34	**0.29**	**0.020**
increaser	PL_SAR	Peary Land	*Salix arctica*	0.95	1.18	0.23	0.088
increaser	RE_SAR	Resolute	*Salix arctica*	1.13	1.02	-0.12	0.357
increaser	TL_BNA	Toolik Lake	*Betula nana*	0.97	1.03	0.06	0.555
increaser	UM_BGL	Umiujuaq	*Betula glandulosa*	0.90	1.18	**0.28**	**0.003**
increaser	ZA_SAR	Zackenberg	*Salix arctica*	0.88	0.93	0.05	0.563
			mean	0.91	1.15	**0.24**	**0.000**
decreaser	BL_BGL	Baker Lake	*Betula glandulosa*	1.20	0.91	**-0.30**	**0.006**
decreaser	DE_SGL	Dempster	*Salix glauca*	1.09	0.94	-0.15	0.074
decreaser	HE_SAR	Herschel Island	*Salix arctica*	1.14	0.97	-0.17	0.328
decreaser	HE_SRI	Herschel Island	*Salix richardsonii*	1.18	0.80	**-0.38**	**0.000**
decreaser	KG_BNA	Kangerlussuaq	*Betula nana*	1.16	0.95	**-0.21**	**0.036**
decreaser	KG_SGL	Kangerlussuaq	*Salix glauca*	1.07	0.94	-0.13	0.198
decreaser	KY_BNA	Kytalyk Reserve	*Betula nana*	0.97	0.64	**-0.33**	**0.000**
decreaser	KY_SPU	Kytalyk Reserve	Salix pulchra	1.05	0.70	**-0.35**	**0.007**
decreaser	VA_SRI	Varandei	*Salix lanata*	1.21	0.98	-0.23	0.136
			mean	1.12	0.87	**-0.25**	**0.000**

Table S4. Changes in mean summer (June-July-August) temperature, precipitation and SPEI for each study site for the study period (1979-2008), as well for early (1979-1993) and recent (1994-2008) period calculated using linear regression. Values marked in bold indicate a statistically significant changes in analyzed climatic parameters (*p*<0.05). Climatic data sources: see Table S9.

		TEMP JJA						PRECIP JJA						SPEI JJA					
Responder	Site ID	(1979-2008)	(1979-1993)	(1994-2008)	(1979-2008)			(1979-2008)	(1979-1993)	(1994-2008)	(1979-2008)			(1979-2008)	(1979-1993)	(1994-2008)	(1979-2008)		
		[ºC]	[ºC]	[ºC]	β	p-value	R2	[mm]	[mm]	[mm]	β	p-value	R2				β	p-value	R2
increaser	AF	0.8	0.5	1.0	**0.05**	**0.006**	**0.237**	77	70	85	0.74	0.323	0.035	-0.443	-0.283	-0.596	**-0.026**	**0.008**	**0.227**
increaser	BS	8.1	7.5	8.7	**0.07**	**0.001**	**0.321**	185	192	177	-1.20	0.266	0.044	1.353	1.406	1.286	-0.006	0.360	0.030
increaser	BT	8.1	7.5	8.7	**0.07**	**0.001**	**0.321**	185	192	177	-1.20	0.266	0.044	1.315	1.406	1.286	0.001	0.881	0.001
increaser	DK	4.4	4.3	4.6	0.02	0.120	0.084	92	92	91	0.18	0.745	0.004	-0.048	0.010	-0.105	-0.001	0.869	0.001
increaser	EB	2.6	2.2	2.9	**0.06**	**0.000**	**0.487**	100	93	107	0.35	0.543	0.013	0.732	0.971	0.510	**-0.029**	**0.001**	**0.352**
increaser	GR	8.1	7.8	8.5	**0.04**	**0.008**	**0.226**	218	185	250	**3.78**	**0.000**	**0.406**	1.594	1.455	1.749	**0.020**	**0.002**	**0.304**
increaser	LA	-1.1	-1.3	-0.9	**0.04**	**0.015**	**0.194**	53	53	53	-0.03	0.953	0.000	-0.341	-0.201	-0.488	**-0.030**	**0.016**	**0.190**
increaser	LB	10.7	10.5	10.8	0.04	0.094	0.097	150	152	148	-0.54	0.419	0.023	-0.023	-0.012	-0.068	**-0.010**	**0.039**	**0.143**
increaser	PL	-1.9	-2.1	-1.7	**0.04**	**0.011**	**0.208**	29	30	28	-0.36	0.254	0.046	-0.760	-0.554	-1.067	**-0.083**	**0.009**	**0.222**
increaser	RE	2.1	1.9	2.4	**0.04**	**0.039**	**0.144**	71	67	75	0.17	0.750	0.004	-0.611	-0.601	-0.627	-0.009	0.292	0.040
increaser	TL	9.8	9.6	10.0	0.03	0.060	0.120	103	117	89	**-1.61**	**0.006**	**0.236**	-1.013	-0.867	-1.149	**-0.019**	**0.000**	**0.470**
increaser	UM	8.8	8.2	9.3	**0.07**	**0.001**	**0.326**	203	206	200	-0.66	0.527	0.014	1.489	1.563	1.399	-0.010	0.146	0.074
increaser	ZA	2.7	2.4	3.0	**0.05**	**0.000**	**0.421**	60	68	51	-0.83	0.222	0.053	-0.101	-0.012	-0.194	-0.015	0.096	0.096
	mean	4.9	4.5	5.2	0.05	0.027	0.245	117.3	116.8	117.8	-0.09	0.406	0.071	0.242	0.329	0.149	-0.017	0.209	0.165
decreaser	BL	8.7	8.1	9.4	**0.06**	**0.010**	**0.214**	116	117	115	-0.28	0.729	0.004	-0.060	0.059	-0.244	**-0.018**	**0.004**	**0.259**
decreaser	DE	8.0	7.9	8.0	0.01	0.794	0.002	103	102	103	-0.17	0.767	0.003	-0.605	-0.549	-0.632	-0.002	0.535	0.014
decreaser	HE	8.6	8.4	8.7	0.01	0.494	0.017	63	83	44	**-2.11**	**0.000**	**0.411**	-0.973	-0.823	-1.185	**-0.027**	**0.000**	**0.535**
decreaser	KG	8.6	8.3	8.9	**0.03**	**0.020**	**0.178**	94	92	97	0.32	0.495	0.017	-0.494	-0.482	-0.549	-0.006	0.240	0.049
decreaser	KY	8.7	8.3	9.1	**0.06**	**0.008**	**0.226**	80	92	67	-1.05	0.143	0.075	-0.756	-0.652	-0.787	-0.003	0.686	0.006
decreaser	VA	10.4	10.2	10.5	0.05	0.101	0.093	141	140	141	-0.05	0.937	0.000	0.250	0.208	0.305	-0.001	0.870	0.001
	mean	8.8	8.5	9.1	0.04	0.238	0.122	99.5	104.4	94.6	-0.56	0.512	0.085	-0.440	-0.373	-0.515	-0.010	0.389	0.144

Table S5. Pearson's correlation coefficients between standardized shrub chronologies and monthly (from previous June to current August) Pan-Arctic SIE (raw data) for the study period (1979-2008). Significant values (*p*<0.05) are indicated in black, whereas bold values represent significant coefficients obtained with P-values adjusted for the false discovery rate. Blue cells - *increasers*; red cells - *decreasers*.

Chronology ID	pJun	pJul	pAug	pSep	pOct	pNov	pDec	JAN	FEB	MAR	APR	MAY	JUN	JUL	AUG
AF_SAR	-0.124	-0.361	-0.457	-0.439	**-0.544**	-0.209	-0.178	-0.074	-0.057	-0.020	-0.003	0.040	-0.110	-0.183	-0.260
BL_BGL	**0.654**	**0.492**	**0.540**	**0.415**	**0.414**	**0.399**	**0.552**	**0.618**	**0.602**	**0.608**	**0.645**	**0.613**	**0.582**	**0.555**	**0.549**
BS_BGL	**-0.531**	**-0.654**	**-0.645**	**-0.715**	**-0.783**	**-0.657**	**-0.683**	**-0.635**	**-0.570**	**-0.509**	**-0.423**	-0.315	**-0.634**	**-0.602**	**-0.561**
BT_BGL	-0.302	-0.356	-0.210	-0.255	-0.314	-0.367	-0.295	-0.334	-0.231	-0.285	-0.232	-0.100	-0.380	-0.350	-0.184
DE_SGL	**0.512**	**0.489**	**0.508**	**0.566**	**0.506**	**0.410**	**0.599**	**0.555**	**0.569**	**0.456**	**0.466**	**0.435**	**0.595**	**0.528**	**0.546**
DK_BNA	**-0.461**	**-0.451**	-0.310	-0.304	**-0.501**	**-0.645**	**-0.446**	**-0.466**	-0.371	**-0.415**	-0.342	-0.114	-0.366	**-0.515**	**-0.444**
EB_SPO	-0.171	-0.073	-0.045	-0.059	-0.082	-0.172	-0.295	-0.207	-0.204	-0.088	-0.265	-0.249	-0.406	-0.386	-0.281
GR_BGL	-0.343	-0.306	-0.431	-0.480	-0.397	-0.408	-0.439	-0.386	-0.377	-0.305	-0.159	-0.158	-0.313	-0.364	-0.403
HE_SAR	**0.594**	**0.446**	**0.522**	**0.455**	**0.428**	**0.368**	**0.554**	**0.600**	**0.675**	**0.597**	**0.617**	**0.640**	**0.573**	**0.568**	**0.689**
HE_SRI	**0.709**	**0.620**	**0.688**	**0.682**	**0.552**	**0.630**	**0.746**	**0.801**	**0.803**	**0.743**	**0.739**	**0.743**	**0.795**	**0.692**	**0.664**
KG_BNA	0.344	0.364	**0.398**	**0.456**	**0.482**	**0.559**	**0.522**	**0.560**	**0.522**	**0.473**	**0.404**	0.359	**0.405**	0.327	0.343
KG_SGL	0.250	0.348	**0.418**	**0.467**	**0.428**	0.346	**0.387**	**0.486**	**0.523**	**0.462**	**0.442**	**0.445**	0.361	0.170	0.292
KY_BNA	**0.493**	**0.495**	**0.373**	**0.374**	**0.447**	**0.487**	**0.482**	**0.554**	**0.536**	**0.576**	**0.590**	**0.427**	**0.480**	**0.376**	**0.424**
KY_SPU	**0.511**	**0.540**	**0.453**	**0.460**	**0.398**	**0.507**	**0.385**	**0.473**	**0.449**	**0.491**	**0.544**	**0.384**	0.320	0.303	0.309
LA_SAR	-0.044	-0.186	-0.173	-0.246	-0.444	-0.134	-0.113	-0.008	-0.037	0.039	0.116	0.213	-0.154	-0.223	-0.250
LB_SRI	**-0.493**	-0.374	**-0.517**	**-0.470**	-0.307	-0.279	-0.316	**-0.448**	**-0.558**	**-0.548**	**-0.583**	**-0.609**	**-0.539**	**-0.475**	**-0.547**
PL_SAR	-0.321	-0.309	-0.367	-0.338	**-0.517**	**-0.565**	**-0.528**	-0.319	-0.300	-0.192	-0.335	-0.320	-0.369	-0.397	**-0.491**
RE_SAR	-0.179	-0.138	-0.130	-0.062	-0.083	-0.126	-0.164	-0.031	-0.083	-0.116	-0.121	-0.072	0.019	-0.193	-0.325
TL_BNA	-0.105	-0.199	-0.239	-0.277	-0.188	-0.322	-0.144	-0.175	-0.156	-0.198	-0.210	-0.262	-0.261	-0.248	-0.215
UM_BGL	**-0.483**	-0.308	-0.386	-0.373	-0.380	**-0.434**	**-0.478**	**-0.541**	**-0.490**	**-0.415**	**-0.397**	-0.326	**-0.634**	**-0.598**	**-0.493**
VA_SRI	0.285	0.454	0.265	0.290	**0.500**	**0.562**	**0.491**	0.281	0.093	0.112	0.172	0.018	0.131	0.140	0.109
YR_SRI	-0.099	-0.045	-0.234	-0.181	-0.052	0.143	0.016	-0.010	-0.150	-0.121	-0.164	-0.266	-0.165	-0.116	-0.183
ZA_SAR	-0.089	-0.315	-0.373	-0.398	-0.463	-0.165	-0.128	-0.098	-0.065	-0.020	-0.031	0.028	-0.090	-0.075	-0.156

Table S6. Pearson's correlation coefficients between standardized shrub chronologies and monthly (from previous June to current August) Pan-Arctic SIE (raw data) for the maximum chronology timespan (1979-max. 2016, see Table S1). Significant values (p<0.05) are indicated in black, whereas bold values represent significant coefficients obtained with P-values adjusted for the false discovery rate. Blue cells - *increasers*; red cells - *decreasers*.

Chronology ID	pJun	pJul	pAug	pSep	pOct	pNov	pDec	JAN	FEB	MAR	APR	MAY	JUN	JUL	AUG
AF_SAR	-0.124	-0.361	-0.457	-0.439	**-0.544**	-0.209	-0.178	-0.074	-0.057	-0.020	-0.003	0.040	-0.110	-0.183	-0.260
BL_BGL	**0.701**	**0.559**	**0.610**	**0.513**	**0.492**	**0.462**	**0.617**	**0.665**	**0.655**	**0.639**	**0.637**	**0.607**	**0.638**	**0.622**	**0.614**
BS_BGL	**-0.551**	**-0.667**	**-0.656**	**-0.711**	**-0.791**	**-0.667**	**-0.694**	**-0.648**	**-0.587**	**-0.525**	**-0.432**	-0.316	**-0.648**	**-0.619**	**-0.579**
BT_BGL	-0.288	-0.344	-0.196	-0.229	-0.308	-0.361	-0.281	-0.327	-0.226	-0.281	-0.231	-0.101	-0.370	-0.338	-0.178
DE_SGL	**0.512**	**0.489**	**0.508**	**0.566**	**0.506**	**0.410**	**0.599**	**0.555**	**0.569**	**0.456**	**0.466**	**0.435**	**0.595**	**0.528**	**0.546**
DK_BNA	**-0.554**	**-0.561**	**-0.485**	**-0.498**	**-0.620**	**-0.678**	**-0.557**	**-0.567**	**-0.510**	**-0.486**	**-0.358**	-0.194	**-0.549**	**-0.628**	**-0.569**
EB_SPO	-0.179	-0.090	-0.065	-0.077	-0.101	-0.183	-0.295	-0.218	-0.214	-0.102	-0.269	-0.256	-0.385	-0.375	-0.283
GR_BGL	-0.171	-0.171	-0.275	-0.289	-0.247	-0.272	-0.240	-0.231	-0.231	-0.188	-0.096	-0.099	-0.158	-0.189	-0.241
HE_SAR	**0.594**	**0.446**	**0.522**	**0.455**	**0.428**	**0.368**	**0.554**	**0.600**	**0.675**	**0.597**	**0.617**	**0.640**	**0.573**	**0.568**	**0.689**
HE_SRI	**0.709**	**0.620**	**0.688**	**0.682**	**0.552**	**0.630**	**0.746**	**0.801**	**0.803**	**0.743**	**0.739**	**0.743**	**0.795**	**0.692**	**0.664**
KG_BNA	**0.574**	**0.564**	**0.573**	**0.616**	**0.638**	**0.687**	**0.686**	**0.694**	**0.675**	**0.605**	**0.474**	**0.458**	**0.614**	**0.578**	**0.556**
KG_SGL	**0.376**	**0.416**	**0.470**	**0.513**	**0.467**	**0.434**	**0.488**	**0.550**	**0.587**	**0.543**	**0.490**	**0.482**	**0.454**	0.332	**0.370**
KY_BNA	0.350	0.373	0.218	0.181	0.354	0.402	0.341	0.456	0.427	**0.488**	**0.530**	0.399	0.376	0.263	0.310
KY_SPU	**0.511**	**0.540**	**0.453**	**0.460**	**0.398**	**0.507**	**0.385**	**0.473**	**0.449**	**0.491**	**0.544**	0.384	0.320	0.303	0.309
LA_SAR	-0.044	-0.186	-0.173	-0.246	-0.444	-0.134	-0.113	-0.008	-0.037	0.039	0.116	0.213	-0.154	-0.223	-0.250
LB_SRI	**-0.493**	-0.374	**-0.517**	**-0.470**	-0.307	-0.279	-0.316	**-0.448**	**-0.558**	**-0.548**	**-0.583**	**-0.609**	**-0.539**	**-0.475**	**-0.547**
PL_SAR	-0.186	-0.122	-0.149	-0.144	-0.287	-0.374	-0.332	-0.209	-0.193	-0.139	-0.249	-0.228	-0.274	-0.226	-0.291
RE_SAR	-0.179	-0.138	-0.130	-0.062	-0.083	-0.126	-0.164	-0.031	-0.083	-0.116	-0.121	-0.072	0.019	-0.193	-0.325
TL_BNA	-0.320	-0.303	-0.353	-0.378	-0.379	-0.401	-0.304	-0.260	-0.228	-0.174	-0.138	-0.180	-0.306	-0.370	-0.329
UM_BGL	**-0.413**	-0.254	-0.307	-0.277	-0.309	**-0.387**	**-0.401**	**-0.479**	**-0.426**	**-0.382**	**-0.384**	-0.315	**-0.518**	**-0.505**	**-0.422**
VA_SRI	0.285	0.454	0.265	0.290	**0.500**	**0.562**	**0.491**	0.281	0.093	0.112	0.172	0.018	0.131	0.140	0.109
YR_SRI	-0.100	-0.048	-0.225	-0.171	-0.055	0.138	0.008	-0.015	-0.150	-0.123	-0.165	-0.266	-0.166	-0.118	-0.182
ZA_SAR	-0.121	-0.336	-0.360	-0.355	-0.506	-0.222	-0.168	-0.163	-0.135	-0.059	-0.033	-0.021	-0.211	-0.158	-0.208

Table S7. Pearson's correlation coefficients between standardized shrub chronologies and monthly (from previous June to current August) regional SIE (raw data) for common period (1979-2008). Significant values (p<0.05) are indicated in black, whereas bold values represent significant coefficients obtained with P-values adjusted for the false discovery rate. Blue cells - *increasers*; red cells - *decreasers*.

Chronology ID	pJun	pJul	pAug	pSep	pOct	pNov	pDec	JAN	FEB	MAR	APR	MAY	JUN	JUL	AUG
AF_SAR	0.000	-0.160	-0.166	-0.157	-0.086	-0.025	0.074	0.187	0.154	0.190	0.163	-0.014	-0.091	-0.073	-0.120
BL_BGL	0.222	0.221	0.251	0.255	0.445	0.311	0.108	0.270	0.276	0.046	0.219	0.397	0.238	0.422	0.476
BS_BGL	**-0.522**	**-0.525**	**-0.492**	**-0.411**	**-0.495**	**-0.532**	**-0.471**	-0.173	-0.277	-0.199	-0.030	-0.347	**-0.701**	**-0.610**	**-0.551**
BT_BGL	**-0.538**	**-0.408**	-0.369	**-0.424**	**-0.453**	**-0.479**	**-0.546**	-0.300	0.129	-0.090	-0.064	**-0.466**	**-0.706**	**-0.520**	**-0.437**
DE_SGL	0.021	-0.006	0.087	0.321	0.145	0.026	0.276	-0.335	0.096	0.260	0.002	-0.089	-0.134	-0.134	0.033
DK_BNA	**-0.522**	-0.350	-0.142	0.132	-0.122	**-0.497**	**-0.647**	**-0.564**	**-0.484**	**-0.464**	**-0.490**	**-0.452**	**-0.529**	**-0.571**	-0.405
EB_SPO	-0.121	-0.263	-0.256	-0.284	-0.404	-0.268	-0.264	-0.189	-0.203	-0.131	-0.031	-0.067	-0.032	-0.152	-0.265
GR_BGL	-0.269	-0.146	-0.259	-0.189	-0.284	-0.322	-0.304	-0.091	-0.167	-0.215	0.214	-0.097	-0.264	-0.317	-0.357
HE_SAR	0.015	0.004	-0.038	0.168	0.184	0.023	0.345	-0.024	0.143	0.336	0.089	-0.210	-0.105	0.062	0.156
HE_SRI	-0.029	-0.061	0.018	0.373	0.172	-0.038	0.239	-0.013	0.282	0.164	0.245	0.068	0.086	0.077	0.078
KG_BNA	**0.603**	**0.537**	0.275	0.178	**0.390**	**0.439**	**0.498**	**0.415**	**0.503**	**0.505**	**0.481**	**0.485**	**0.493**	**0.463**	0.192
KG_SGL	0.392	0.456	0.378	0.336	0.511	0.357	0.293	0.173	0.132	0.195	0.232	0.226	0.274	0.271	0.085
KY_BNA	-0.251	-0.184	0.040	0.156	0.184	0.164	0.129	-0.246	0.084	-0.117	-0.051	-0.063	-0.083	-0.092	0.112
KY_SPU	0.053	0.096	0.286	0.410	0.450	0.353	0.268	-0.373	0.143	-0.171	-0.064	-0.044	-0.054	-0.091	0.117
LA_SAR	0.241	0.118	-0.168	-0.441	-0.443	-0.363	-0.269	-0.130	-0.160	-0.075	0.170	0.171	0.146	0.213	0.032
LB_SRI	-0.122	-0.103	-0.021	0.168	0.070	-0.010	-0.091	-0.227	-0.338	0.285	-0.012	0.040	-0.158	-0.366	-0.193
PL_SAR	-0.076	-0.160	-0.237	-0.297	-0.313	-0.262	-0.218	0.121	0.004	-0.048	0.295	0.143	0.274	0.265	-0.069
RE_SAR	0.045	-0.136	-0.225	-0.200	-0.414	-0.004	-0.050	0.068	0.046	0.026	0.009	-0.093	-0.101	-0.205	-0.443
TL_BNA	-0.165	-0.151	-0.298	-0.376	-0.437	-0.170	-0.121	-0.027	-0.005	-0.114	0.078	-0.266	-0.339	-0.482	-0.480
UM_BGL	-0.066	0.020	-0.210	-0.196	-0.314	-0.347	-0.118	-0.275	-0.230	-0.111	-0.018	-0.313	**-0.548**	**-0.576**	**-0.473**
VA_SRI	-0.019	0.212	0.417	0.405	0.481	0.400	0.257	0.005	-0.191	-0.069	-0.124	-0.151	-0.049	0.020	0.207
YR_SRI	-0.082	-0.012	-0.026	0.071	-0.034	0.095	0.191	-0.118	-0.150	0.019	-0.122	0.071	-0.031	-0.170	-0.043
ZA_SAR	0.030	0.008	0.149	0.223	0.151	0.102	0.026	-0.042	-0.071	-0.060	0.144	0.250	0.024	-0.150	-0.186

Table S8. Output of linear regression models between standardized shrub chronologies and seasonal Pan-Arctic and regional SIE (all data z-score) for common period (1979-2008). List of predictors and data sources are presented in Table S9. Significant values (p<0.05) indicated in bold. Blue font - *increasers*; red font - *decreasers*. Grey font - non-significant values.

Chronology ID	JJA SIE						pSON SIE						DJF SIE						MAM SIE					
	Pan-Arctic			regional			Pan-Arctic			regional			Pan-Arctic			regional			Pan-Arctic			regional		
	R^2	β	*p*-value	R^2	β	*p*-value	R^2	β	*p*-value	R^2	β	*p*-value	R^2	β	*p*-value	R^2	β	*p*-value	R^2	β	*p*-value	R^2	β	*p*-value
AF_SAR	0.043	-0.078	0.272	0.011	-0.034	0.578	**0.191**	**-0.165**	**0.018**	0.012	-0.033	0.578	0.012	-0.036	0.578	0.022	0.043	0.439	0.000	0.002	0.972	0.014	0.032	0.537
BL_BGL	**0.341**	**0.243**	**0.001**	**0.173**	**0.141**	**0.022**	0.194	**0.184**	**0.017**	**0.146**	**0.131**	**0.040**	**0.203**	**0.168**	**0.014**	0.070	0.159	0.165	**0.425**	**0.226**	**0.000**	0.116	0.205	0.065
BS_BGL	**0.382**	**-0.429**	**0.000**	**0.466**	**-0.386**	**0.000**	**0.599**	**-0.533**	**0.000**	**0.295**	**-0.306**	**0.002**	**0.373**	**-0.375**	**0.000**	**0.213**	**-0.457**	**0.012**	**0.194**	**-0.255**	**0.015**	0.075	-0.275	0.143
BT_BGL	0.091	-0.246	0.105	**0.372**	**-0.406**	**0.000**	0.107	-0.261	0.083	**0.253**	**-0.329**	**0.005**	0.073	-0.193	0.155	0.042	-0.234	0.289	0.048	-0.150	0.243	0.085	-0.344	0.119
DE_SGL	**0.329**	**0.181**	**0.001**	0.009	-0.026	0.623	0.292	**0.172**	**0.002**	0.031	0.070	0.362	**0.306**	**0.157**	**0.002**	0.000	0.003	0.960	**0.228**	**0.126**	**0.008**	0.046	0.128	0.253
DK_BNA	**0.223**	**-0.274**	**0.008**	**0.303**	**-0.272**	**0.002**	**0.247**	**-0.286**	**0.006**	0.028	-0.078	0.390	**0.205**	**-0.233**	**0.014**	**0.386**	**-0.275**	**0.000**	0.095	-0.150	0.097	**0.239**	**-0.206**	**0.006**
EB_SPO	**0.131**	**-0.184**	**0.049**	0.029	-0.077	0.367	0.011	-0.054	0.583	0.112	-0.137	0.076	0.030	-0.079	0.365	0.039	-0.085	0.305	0.044	-0.089	0.266	0.007	-0.036	0.671
GR_BGL	**0.148**	**-0.132**	**0.039**	0.118	-0.094	0.068	**0.218**	**-0.158**	**0.011**	0.092	-0.084	0.109	**0.242**	**-0.148**	**0.007**	0.090	-0.146	0.114	0.049	-0.065	0.250	0.000	-0.007	0.945
HE_SAR	**0.416**	**0.408**	**0.000**	0.001	0.017	0.874	**0.206**	**0.278**	**0.013**	0.018	0.101	0.493	**0.257**	**0.278**	**0.005**	0.047	0.131	0.259	**0.419**	**0.341**	**0.000**	0.059	0.289	0.195
HE_SRI	**0.537**	**0.306**	**0.000**	0.007	0.032	0.653	**0.454**	**0.255**	**0.000**	0.028	0.079	0.387	**0.542**	**0.248**	**0.000**	0.057	0.090	0.210	**0.604**	**0.270**	**0.000**	0.038	0.153	0.301
KG_BNA	**0.133**	**0.137**	**0.047**	**0.174**	**0.133**	**0.022**	**0.281**	**0.201**	**0.003**	**0.146**	**0.118**	**0.041**	**0.343**	**0.198**	**0.001**	**0.248**	**0.145**	**0.006**	**0.189**	**0.136**	**0.016**	**0.261**	**0.140**	**0.004**
KG_SGL	0.074	0.103	0.145	0.052	0.073	0.225	**0.206**	**0.168**	**0.014**	**0.195**	**0.133**	**0.017**	**0.220**	**0.154**	**0.010**	0.057	0.068	0.211	**0.223**	**0.149**	**0.008**	0.051	0.062	0.228
KY_BNA	**0.190**	**0.142**	**0.016**	0.001	-0.007	0.892	**0.212**	**0.143**	**0.012**	0.033	0.056	0.342	**0.234**	**0.133**	**0.008**	0.003	-0.022	0.764	**0.314**	**0.152**	**0.001**	0.013	-0.036	0.549
KY_SPU	0.104	0.157	0.082	0.000	-0.005	0.952	**0.237**	**0.234**	**0.007**	**0.195**	**0.211**	**0.016**	**0.276**	**0.226**	**0.003**	0.002	0.024	0.836	**0.247**	**0.201**	**0.005**	0.019	-0.064	0.469
LA_SAR	0.052	-0.132	0.224	0.024	0.132	0.410	0.088	-0.173	0.118	**0.222**	**-0.352**	**0.010**	0.006	-0.039	0.699	0.055	-0.260	0.223	0.015	0.060	0.514	0.029	0.123	0.366
LB_SRI	**0.294**	**-0.285**	**0.003**	0.070	-0.145	0.174	**0.165**	**-0.241**	**0.036**	0.016	0.075	0.526	**0.185**	**-0.186**	**0.025**	0.048	-0.408	0.270	**0.372**	**-0.239**	**0.001**	0.001	0.018	0.863
PL_SAR	**0.199**	**-0.217**	**0.013**	0.033	0.129	0.336	**0.243**	**-0.241**	**0.007**	0.106	-0.205	0.084	0.128	-0.156	0.057	0.001	-0.022	0.903	0.088	-0.120	0.112	0.056	0.142	0.209
RE_SAR	0.043	-0.096	0.272	0.079	-0.123	0.132	0.009	-0.044	0.627	0.059	-0.113	0.203	0.003	-0.021	0.789	0.002	0.017	0.827	0.012	-0.042	0.572	0.002	-0.025	0.835
TL_BNA	0.061	-0.094	0.187	**0.210**	**-0.153**	**0.011**	0.079	-0.106	0.139	0.133	-0.170	0.052	0.011	-0.035	0.589	0.004	-0.022	0.757	0.054	-0.073	0.217	0.047	-0.153	0.252
UM_BGL	**0.346**	**-0.215**	**0.001**	**0.343**	**-0.175**	**0.001**	**0.179**	**-0.151**	**0.022**	0.093	-0.089	0.108	**0.231**	**-0.153**	**0.008**	0.047	-0.111	0.259	**0.160**	**-0.122**	**0.028**	0.042	-0.109	0.277
VA_SRI	0.015	0.083	0.545	0.004	0.033	0.744	**0.236**	**0.378**	**0.012**	**0.233**	**0.246**	**0.013**	0.099	0.183	0.117	0.000	0.008	0.956	0.010	0.053	0.613	0.019	-0.075	0.498
YR_SRI	0.026	-0.064	0.394	0.008	-0.043	0.628	0.003	-0.021	0.785	0.002	0.021	0.825	0.001	0.012	0.860	0.000	-0.012	0.949	0.036	-0.063	0.314	0.001	-0.019	0.838
ZA_SAR	0.013	-0.033	0.541	0.014	-0.031	0.527	**0.143**	**-0.109**	**0.043**	0.030	0.041	0.369	0.015	-0.031	0.529	0.000	-0.004	0.927	0.000	-0.002	0.960	0.015	0.030	0.526

Table S9. List of sea-ice and climate-related predictors used in SEM and analyses and in LME models (fixed effects) together with data sources. Climate variables (temperature, precipitation, SPEI) were calculated from CRU TS 4.01 data. SPEI was calculated using spei package (v1.7) (13, 14) in R. All predictors were standardized before the analyses (z-score), except for SPEI which was already represented by an index. Response variable was represented by ring-width index (RWI), which was a standardized mean annual growth after diameter RCS detrending (i.e., each chronology per responder group for SEM analyses and each shrub for LME models).

Predictor type	Variable code	Definition	Data Source
Pan-Arctic sea-ice extent (SIE)	pSON_pA	previous September-October-November mean Pan-Arctic sea-ice extent	NOAA/NSIDC (Climate Data Record of Passive Microwave Sea IceConcentration, Version 2)
	DJF_pA	previous December-January-February mean Pan-Arctic sea-ice extent	NOAA/NSIDC (Climate Data Record of Passive Microwave Sea IceConcentration, Version 2)
	MAM_pA	March-April-May mean Pan-Arctic sea-ice extent	NOAA/NSIDC (Climate Data Record of Passive Microwave Sea IceConcentration, Version 2)
	JJA_pA	June-July-August mean Pan-Arctic sea-ice extent	NOAA/NSIDC (Climate Data Record of Passive Microwave Sea IceConcentration, Version 2)
Regional sea-ice extent (SIE)	pSON	previous September-October-November mean regional sea-ice extent	NOAA/NSIDC (Climate Data Record of Passive Microwave Sea IceConcentration, Version 3)
	DJF	previous December-January-February mean regional sea-ice extent	NOAA/NSIDC (Climate Data Record of Passive Microwave Sea IceConcentration, Version 3)
	MAM	March-April-May mean regional sea-ice extent	NOAA/NSIDC (Climate Data Record of Passive Microwave Sea IceConcentration, Version 3)
	JJA	June-July-August mean regional sea-ice extent	NOAA/NSIDC (Climate Data Record of Passive Microwave Sea IceConcentration, Version 3)
Timing of regional sea-ice retreat and advance	pFAD	previous year first advance day of sea-ice at 50% sea-ice concentration threshold	Stroeve et al. (2016)
	pLRD	previous year last retreat day of regional sea-ice at 50% sea-ice concentration threshold	Stroeve et al. (2016)
	LRD	last retreat day of regional sea-ice at 50% sea-ice concentration threshold	Stroeve et al. (2016)
Climate variables	TEMP_JJA	June-July-August mean temperature	CRU TS 4.01
	PRECIP_JJA	June-July-August precipitation sum	CRU TS 4.01
	SPEI_JJA	June-July-August mean standardized precipitation evaporation index	CRU TS 4.01, spei package

Table S10. Piecewise SEM results for *increasers*. Linear mixed effect (LME) models for RWI, summer (June-to-August, JJA) SPEI, temperature and precipitation were implemented into each SEM with a random intercept for a site included in each model. Models were run for period (1979-2008), except for previous year sea ice variables (i.e. pLRD, pFAD, DJF, pSON, DJF_pA, pSON_pA) for which we lack measurements for year 1979. For the source data and the abbreviations, see Table S9.

increasers

Sea ice variable included in the model		p-value	Fisher's C test		Standardized parameter estimates: RWI			SPEI JJA			PRECIP JJA		TEMP JJA	R^2 endogenous variables				AIC	AICc
	Variable code				Sea ice variable	TEMP JJA	SPEI JJA	Sea ice variable	PRECIP JJA	TEMP JJA	Sea ice variable	TEMP JJA	Sea ice variable	RWI	SPEI JJA	PRECIP JJA	TEMP JJA		
regional sea ice variables	LRD	0.084	4.957	Est.	-0.064	**0.425**	-0.035	-0.047	**0.056**	**-0.230**	**-0.209**	**-0.208**	**-0.463**	0.27	0.78	0.05	0.21	46.96	49.80
				SE	0.021	0.020	0.024	0.032	0.027	0.030	0.060	0.056	0.048						
				df	370	370	370	373	373	373	375	375	376						
				p-value	0.195	**0.000**	0.591	0.096	**0.028**	**0.000**	**0.000**	**0.000**	**0.000**						
	pLRD	0.331	2.214	Est.	**-0.130**	**0.427**	-0.040	-0.020	**0.066**	**-0.210**	0.051	-0.077	**-0.196**	0.29	0.78	0.01	0.04	44.21	46.97
				SE	0.019	0.019	0.025	0.029	0.027	0.028	0.055	0.053	0.053						
				df	359	359	359	361	361	361	362	362	363						
				p-value	**0.004**	**0.000**	0.556	0.424	**0.008**	**0.000**	0.335	0.146	**0.000**						
	pFAD	0.194	3.28	Est.	**0.145**	**0.415**	-0.040	0.004	**0.065**	**-0.208**	0.055	-0.100	**0.257**	0.30	0.78	0.01	0.07	45.28	48.10
				SE	0.020	0.019	0.025	0.031	0.027	0.028	0.058	0.054	0.054						
				df	359	359	359	361	361	361	362	362	363						
				p-value	**0.002**	**0.000**	0.558	0.879	**0.010**	**0.000**	0.306	0.060	**0.000**						
	JJA	0.058	5.707	Est.	**-0.122**	**0.391**	-0.035	-0.017	**0.061**	**-0.216**	**-0.196**	**-0.214**	**-0.523**	0.28	0.78	0.04	0.27	47.71	50.59
				SE	0.025	0.021	0.024	0.039	0.027	0.032	0.072	0.058	0.054						
				df	370	370	370	373	373	373	375	375	376						
				p-value	**0.018**	**0.000**	0.586	0.574	**0.016**	**0.000**	**0.001**	**0.000**	**0.000**						
	MAM	0.139	3.948	Est.	-0.001	**0.456**	-0.027	0.001	**0.064**	**-0.207**	**-0.144**	**-0.139**	**-0.190**	0.27	0.78	0.03	0.04	45.95	48.73
				SE	0.025	0.018	0.024	0.039	0.027	0.027	0.073	0.051	0.072						
				df	370	370	370	373	373	373	375	375	376						
				p-value	0.978	**0.000**	0.677	0.985	**0.012**	**0.000**	**0.005**	**0.007**	**0.000**						
	DJF	0.191	3.313	Est.	**-0.094**	**0.438**	-0.030	0.023	**0.067**	**-0.202**	-0.084	-0.101	**-0.169**	0.28	0.78	0.01	0.03	45.31	48.14
				SE	0.025	0.019	0.025	0.039	0.027	0.028	0.073	0.053	0.070						
				df	359	359	359	361	361	361	362	362	363						
				p-value	**0.039**	**0.000**	0.661	0.371	**0.008**	**0.000**	0.107	0.054	**0.001**						
	pSON	0.235	2.9	Est.	**-0.155**	**0.410**	-0.031	0.020	**0.066**	**-0.201**	-0.015	-0.091	**-0.282**	0.30	0.78	0.01	0.08	44.90	47.70
				SE	0.022	0.019	0.025	0.034	0.027	0.029	0.065	0.054	0.059						
				df	359	359	359	361	361	361	362	362	363						
				p-value	**0.001**	**0.000**	0.645	0.440	**0.009**	**0.000**	0.779	0.092	**0.000**						
Pan-Arctic sea ice variables	JJA_pA	0.113	4.35	Est.	**-0.123**	**0.391**	-0.017	0.055	**0.065**	**-0.177**	-0.045	**-0.136**	**-0.549**	0.28	0.78	0.01	0.30	46.36	49.16
				SE	0.028	0.021	0.024	0.043	0.027	0.032	0.081	0.060	0.057						
				df	370	370	370	373	373	373	375	375	376						
				p-value	**0.021**	**0.000**	0.790	0.066	**0.009**	**0.000**	0.457	**0.025**	**0.000**						
	MAM_pA	0.147	3.838	Est.	-0.068	**0.436**	-0.023	0.030	**0.063**	**-0.197**	0.016	**-0.106**	**-0.320**	0.27	0.78	0.01	0.10	45.84	48.61
				SE	0.021	0.019	0.024	0.031	0.027	0.028	0.060	0.053	0.054						
				df	370	370	370	373	373	373	375	375	376						
				p-value	0.147	**0.000**	0.726	0.250	**0.011**	**0.000**	0.771	**0.047**	**0.000**						
	DJF_pA	0.195	3.272	Est.	**-0.131**	**0.410**	-0.029	0.016	**0.066**	**-0.201**	-0.062	-0.108	**-0.344**	0.29	0.78	0.01	0.12	45.27	48.09
				SE	0.022	0.020	0.025	0.034	0.027	0.029	0.064	0.055	0.057						
				df	359	359	359	361	361	361	362	362	363						
				p-value	**0.006**	**0.000**	0.663	0.542	**0.009**	**0.000**	0.262	0.050	**0.000**						
	pSON_pA	0.16	3.662	Est.	**-0.209**	**0.374**	-0.016	0.048	**0.068**	**-0.187**	-0.054	-0.108	**-0.399**	0.31	0.78	0.01	0.16	45.66	48.51
				SE	0.025	0.020	0.024	0.039	0.027	0.030	0.074	0.057	0.062						
				df	359	359	359	361	361	361	362	362	363						
				p-value	**0.000**	**0.000**	0.817	0.075	**0.007**	**0.000**	0.339	0.055	**0.000**						

Table S11. Piecewise SEM results for *decreasers*. Linear mixed effect (LME) models for RWI, summer (June-to-August, JJA) SPEI, temperature and precipitation were implemented into each SEM with a random intercept for a site included in each model. Models were run for period (1979-2008), except for previous year sea ice variables (i.e. pLRD, pFAD, DJF, pSON, DJF_pA, pSON_pA) for which we lack measurements for year 1979. For the source data and the abbreviations, see Table S9.

decreasers

Sea ice variable included in the model		p-value	Fisher's C test		Standardized parameter estimates: RWI			SPEI JJA			PRECIP JJA		TEMP JJA	R^2 endogenous variables				AIC	AICc
	Variable code				Sea ice variable	TEMP JJA	SPEI JJA	Sea ice variable	PRECIP JJA	TEMP JJA	Sea ice variable	TEMP JJA	Sea ice variable	RWI	SPEI JJA	PRECIP JJA	TEMP JJA		
regional sea ice variables	LRD	0.495	1.405	Est.	0.130	0.127	**0.235**	**0.075**	**0.241**	**-0.110**	-0.045	-0.060	**-0.528**	0.10	0.76	0.00	0.28	43.41	47.30
				SE	0.026	0.024	0.053	0.019	0.015	0.018	0.077	0.072	0.056						
				df	255	255	255	258	258	258	259	259	260						
				p-value	0.067	0.070	**0.002**	**0.042**	**0.000**	**0.003**	0.537	0.405	**0.000**						
	pLRD	0.472	1.501	Est.	**0.134**	0.076	**0.190**	**0.066**	**0.216**	**-0.191**	-0.016	-0.066	**-0.235**	0.09	0.85	0.00	0.05	43.50	47.56
				SE	0.023	0.022	0.059	0.013	0.012	0.012	0.070	0.066	0.064						
				df	246	246	246	249	249	249	250	250	251						
				p-value	**0.034**	0.234	**0.018**	**0.013**	**0.000**	**0.000**	0.807	0.302	**0.000**						
	pFAD	0.588	1.061	Est.	**-0.282**	0.092	0.147	**-0.096**	**0.208**	**-0.188**	-0.077	-0.047	**0.201**	0.14	0.85	0.01	0.04	43.06	47.08
				SE	0.023	0.021	0.060	0.013	0.011	0.012	0.070	0.065	0.065						
				df	246	246	246	249	249	249	250	250	251						
				p-value	**0.000**	0.134	0.070	**0.000**	**0.000**	**0.000**	0.224	0.459	**0.001**						
	JJA	0.401	1.83	Est.	**0.143**	0.125	**0.223**	**0.125**	**0.247**	**-0.090**	-0.088	-0.078	**-0.473**	0.10	0.76	0.01	0.22	43.83	47.77
				SE	0.027	0.023	0.053	0.019	0.015	0.017	0.080	0.069	0.062						
				df	255	255	255	258	258	258	259	259	260						
				p-value	**0.036**	0.064	**0.003**	**0.000**	**0.000**	**0.011**	0.205	0.260	**0.000**						
	MAM	0.309	2.347	Est.	**0.146**	0.098	**0.234**	0.035	**0.244**	**-0.140**	**-0.154**	-0.079	**-0.274**	0.11	0.75	0.02	0.08	44.35	48.33
				SE	0.029	0.021	0.053	0.022	0.015	0.016	0.086	0.063	0.080						
				df	255	255	255	258	258	258	259	259	260						
				p-value	**0.020**	0.114	**0.002**	0.292	**0.000**	**0.000**	**0.015**	0.211	**0.000**						
	DJF	0.522	1.3	Est.	**0.176**	0.070	**0.173**	0.020	**0.214**	**-0.203**	0.045	-0.055	**-0.165**	0.10	0.84	0.01	0.03	43.30	47.34
				SE	0.027	0.022	0.060	0.016	0.012	0.012	0.082	0.065	0.077						
				df	246	246	246	249	249	249	250	250	251						
				p-value	**0.006**	0.264	**0.033**	0.451	**0.000**	**0.000**	0.478	0.382	**0.008**						
	pSON	0.777	0.504	Est.	**0.297**	0.085	0.151	**0.088**	**0.204**	**-0.193**	0.118	-0.043	**-0.165**	0.15	0.85	0.02	0.03	42.50	46.47
				SE	0.026	0.021	0.059	0.015	0.011	0.012	0.081	0.065	0.077						
				df	246	246	246	249	249	249	250	250	251						
				p-value	**0.000**	0.160	0.061	**0.001**	**0.000**	**0.000**	0.061	0.492	**0.008**						
Pan-Arctic sea ice variables	JJA_pA	0.892	0.229	Est.	**0.502**	**0.223**	0.109	**0.136**	**0.220**	**-0.099**	**0.164**	0.025	**-0.377**	0.28	0.77	0.02	0.14	42.23	46.02
				SE	0.027	0.019	0.051	0.022	0.015	0.016	0.088	0.065	0.076						
				df	255	255	255	258	258	258	259	259	260						
				p-value	**0.000**	**0.000**	0.130	**0.000**	**0.000**	**0.003**	**0.013**	0.699	**0.000**						
	MAM_pA	0.952	0.099	Est.	**0.514**	**0.169**	**0.138**	**0.094**	**0.228**	**-0.126**	0.122	-0.006	**-0.251**	0.32	0.76	0.02	0.06	42.10	45.88
				SE	0.021	0.018	0.049	**0.017**	**0.015**	0.015	0.070	0.063	0.066						
				df	255	255	255	258	258	258	259	259	260						
				p-value	**0.000**	**0.002**	**0.049**	**0.004**	**0.000**	**0.000**	0.052	0.926	**0.000**						
	DJF_pA	0.421	1.732	Est.	**0.490**	0.108	0.095	**0.063**	**0.202**	**-0.196**	**0.211**	-0.025	**-0.175**	0.29	0.85	0.05	0.03	43.73	47.81
				SE	0.022	0.019	0.057	0.014	0.012	0.012	0.074	0.064	0.071						
				df	246	246	246	249	249	249	250	250	251						
				p-value	**0.000**	0.053	0.224	**0.018**	**0.000**	**0.000**	**0.001**	0.681	**0.005**						
	pSON_pA	0.525	1.288	Est.	**0.477**	0.095	0.077	**0.084**	**0.197**	**-0.194**	**0.217**	-0.028	**-0.159**	0.28	0.85	0.05	0.03	43.29	47.32
				SE	0.026	0.019	0.058	0.016	0.012	0.012	0.083	0.063	0.080						
				df	246	246	246	249	249	249	250	250	251						
				p-value	**0.000**	0.094	0.327	**0.001**	**0.000**	**0.000**	**0.001**	0.649	**0.010**						

Table S12. Parameter estimates (β) for sea ice predictors with associated 95% confidence intervals from top ranked linear mixed-effect models for *increasers* (top) and *decreasers* (bottom) in the study period 1980-2008. Model parameter estimates were calculated using maximum likelihood method. Response variable is represented by all shrubs mean ring width index (RWI) after dRCS detrending and square root transformation. Random intercept of a shrub (n=363 *increasers* and n=251 *decreasers*) nested within a site (n=13 *increasers* and n=9 *decreasers*; i.e. six geographic locations for nine *decreasers* chronologies) was included to each model. SE - standard error; df - number of pairs tested; sd - standard deviation for random effects; R^2m - marginal R^2; R^2c - conditional R^2; AICc - Akaike's information criteria with small sample bias adjustment. Nonsignificant relationships are marked in grey font. For the source data and the abbreviations, see Table S9.

increasers

model	Intercept	β	lower CI	upper CI	SE	df	t-value	sd: Site	sd: Shrub in Site	sd: Residual	AICc	delta	weight	predictor p-value	Intercept p-value	R^2m	R^2c
JJA	0.977	-0.061	-0.069	-0.053	0.004	10870	-15.1	0.046	0.172	0.341	6505	0	1	0.000	0.000	0.017	0.229
pSON_pA	0.985	-0.071	-0.082	-0.061	0.005	10870	-13.5	0.047	0.171	0.343	6549	45	0	0.000	0.000	0.018	0.225
LRD	0.973	-0.043	-0.049	-0.036	0.003	10870	-12.5	0.046	0.173	0.343	6574	70	0	0.000	0.000	0.011	0.222
pSON	0.975	-0.045	-0.054	-0.037	0.004	10870	-10.6	0.046	0.172	0.344	6618	114	0	0.000	0.000	0.009	0.219
JJA_pA	0.981	-0.057	-0.068	-0.046	0.006	10870	-10.3	0.046	0.171	0.345	6622	117	0	0.000	0.000	0.011	0.217
pFAD	0.972	0.025	0.017	0.032	0.004	10870	6.6	0.046	0.173	0.345	6685	180	0	0.000	0.000	0.003	0.215
DJF_pA	0.974	-0.031	-0.041	-0.021	0.005	10870	-6.0	0.046	0.172	0.346	6692	188	0	0.000	0.000	0.004	0.213
pLRD	0.97	-0.017	-0.023	-0.010	0.003	10870	-4.9	0.046	0.173	0.346	6704	199	0	0.000	0.000	0.002	0.213
DJF	0.969	-0.016	-0.026	-0.007	0.005	10870	-3.3	0.046	0.173	0.347	6716	211	0	0.001	0.000	0.001	0.211
MAM	0.968	-0.010	-0.020	-0.001	0.005	10870	-2.1	0.046	0.173	0.348	6722	218	0	0.000	0.034	0.000	0.210
null model	0.967	n.a.	0.934	1.000	0.017	10871	57.1	0.046	0.173	0.348	6725	220	0	n.a.	0.000	0.000	0.210
MAM_pA	0.967	-0.002	-0.011	0.007	0.005	10870	-0.4	0.046	0.173	0.348	6727	222	0	0.681	0.000	0.000	0.210

decreasers

model	Intercept	β	lower CI	upper CI	SE	df	t-value	sd: Site	sd: Shrub in Site	sd: Residual	AICc	delta	weight	predictor p-value	Intercept p-value	R^2m	R^2c
DJF_pA	0.933	0.076	0.065	0.087	0.006	7558	13.3	0.024	0.152	0.308	2175	0	1	0.000	0.000	0.031	0.225
MAM_pA	0.943	0.058	0.048	0.069	0.005	7558	11.3	0.027	0.151	0.310	2220	45	0	0.000	0.000	0.019	0.211
pSON_pA	0.933	0.068	0.056	0.079	0.006	7558	11.2	0.024	0.150	0.311	2221	46	0	0.000	0.000	0.019	0.209
pSON	0.943	0.027	0.018	0.035	0.004	7558	6.2	0.033	0.143	0.317	2299	124	0	0.000	0.000	0.004	0.181
JJA_pA	0.94	0.040	0.028	0.052	0.006	7558	6.5	0.028	0.147	0.314	2299	124	0	0.000	0.000	0.006	0.191
MAM	0.946	0.019	0.009	0.029	0.005	7558	3.9	0.031	0.141	0.319	2321	147	0	0.000	0.000	0.002	0.172
pFAD	0.945	-0.014	-0.021	-0.006	0.004	7558	-3.5	0.032	0.142	0.318	2324	150	0	0.001	0.000	0.001	0.174
DJF	0.948	0.016	0.007	0.026	0.005	7558	3.3	0.029	0.142	0.319	2325	151	0	0.001	0.000	0.001	0.171
JJA	0.946	0.013	0.005	0.021	0.004	7558	3.2	0.032	0.142	0.319	2326	151	0	0.001	0.000	0.001	0.172
LRD	0.946	0.008	0.001	0.015	0.004	7558	2.2	0.033	0.141	0.319	2332	157	0	0.029	0.029	0.000	0.171
null model	0.947	n.a.	0.916	0.978	0.016	7559	59.8	0.033	0.140	0.320	2334	160	0	0.000	n.a.	0.000	0.169
pLRD	0.948	-0.001	-0.008	0.006	0.004	7558	-0.3	0.033	0.140	0.320	2336	162	0	0.750	0.000	0.000	0.168

Table S13. Comparison of best sea ice versus best overall linear mixed effect (LME) models fitted to each site separately using all shrubs (n=19,061 ring-width values in total), instead of chronologies, for common period (1980-2008) per site and species. A random intercept for year was included in each model. Response variable was represented by RWI after dRCS detrending and square root transformation. See Table S9 for full names of predictors used in LME models. SE - standard error; ΔAIC - difference between a null model and best model for each site; R^2m - marginal R^2; R^2c - conditional R^2. All predictor variables were transformed to z-scores prior the analysis. Grey font indicates nonsignificant models.

Chronology ID	Responder	Species	# shrubs	Best sea ice MODEL	ΔAIC	β	SE	p-value	R^2m	R^2c	Best MODEL	ΔAIC	β	SE	p-value	R^2m	R^2c
AF_SAR	*increaser*	*Salix arctica*	29	pSON_pA	3	-0.071	0.031	0.030	0.03	0.14	SPEI_JJA	8	-0.151	0.043	0.002	0.05	0.14
BL_BGL	*decreaser*	*Betula glandulosa*	44	MAM_pA	15	0.120	0.025	0.000	0.09	0.21	MAM_pA	15	0.120	0.025	0.000	0.09	0.21
BS_BGL	*increaser*	*Betula glandulosa*	39	pSON_pA	20	-0.242	0.042	0.000	0.19	0.32	pSON_pA	20	-0.242	0.042	0.000	0.19	0.32
BT_BGL	*increaser*	*Betula glandulosa*	76	JJA	10	-0.159	0.042	0.001	0.11	0.32	JJA	10	-0.159	0.042	0.001	0.11	0.32
DE_SGL	*decreaser*	*Salix glauca*	15	pSON_pA	13	0.102	0.023	0.000	0.07	0.09	pSON_pA	13	0.102	0.023	0.000	0.07	0.09
DK_BNA	*increaser*	*Betula nana*	15	DJF	13	-0.135	0.031	0.000	0.12	0.28	DJF	13	-0.135	0.031	0.000	0.12	0.28
EB_SPO	*increaser*	*Salix polaris*	10	JJA_pA	2	-0.112	0.055	0.052	0.04	0.24	TEMP_JJA	11	0.147	0.019	0.000	0.11	0.24
GR_BGL	*increaser*	*Betula glandulosa*	17	DJF_pA	10	-0.093	0.023	0.000	0.08	0.16	DJF_pA	10	-0.093	0.023	0.000	0.08	0.16
HE_SAR	*decreaser*	*Salix arctica*	24	JJA_pA	18	0.229	0.044	0.000	0.09	0.09	JJA_pA	18	0.229	0.044	0.000	0.09	0.09
HE_SRI	*decreaser*	*Salix richardsonii*	17	DJF_pA	27	0.126	0.019	0.000	0.08	0.08	DJF_pA	27	0.126	0.019	0.000	0.08	0.08
KG_BNA	*decreaser*	*Betula nana*	42	DJF_pA	10	0.105	0.028	0.001	0.07	0.21	DJF_pA	10	0.105	0.028	0.001	0.07	0.21
KG_SGL	*decreaser*	*Salix glauca*	32	DJF_pA	7	0.087	0.027	0.003	0.05	0.14	DJF_pA	7	0.087	0.027	0.003	0.05	0.14
KY_BNA	*decreaser*	*Betula nana*	19	MAM_pA	7	0.075	0.024	0.004	0.04	0.11	MAM_pA	7	0.075	0.024	0.004	0.04	0.11
KY_SPU	*decreaser*	*Salix pulchra*	19	not sig.	0	n.a.	n.a.	n.a.	n.a.	n.a.	not sig.	0	n.a.	n.a.	n.a.	n.a.	n.a.
LA_SAR	*increaser*	*Salix arctica*	19	pSON	3	-0.155	0.070	0.035	0.05	0.33	SPEI_JJA	7	-0.199	0.062	0.003	0.10	0.33
LB_SRI	*increaser*	*Salix lanata*	29	MAM_pA	8	-0.109	0.031	0.002	0.09	0.22	TEMP_JJA	30	0.127	0.008	0.000	0.20	0.22
PL_SAR	*increaser*	*Salix arctica*	10	pSON_pA	3	-0.091	0.038	0.024	0.03	0.11	pSON_pA	3	-0.091	0.038	0.024	0.03	0.11
RE_SAR	*increaser*	*Salix arctica*	31	not sig.	0	n.a.	n.a.	n.a.	n.a.	n.a.	PRECIP_JJA	4	-0.068	0.014	0.000	0.02	0.10
TL_BNA	*increaser*	*Betula nana*	29	LRD	6	-0.083	0.028	0.006	0.04	0.12	TEMP_JJA	15	0.094	0.011	0.000	0.07	0.12
UM_BGL	*increaser*	*Betula glandulosa*	25	LRD	7	-0.063	0.020	0.003	0.03	0.07	TEMP_JJA	8	0.054	0.010	0.000	0.04	0.07
VA_SRI	*decreaser*	*Salix lanata*	39	pSON	5	0.126	0.045	0.010	0.11	0.44	TEMP_JJA	16	0.145	0.007	0.000	0.23	0.44
ZA_SAR	*increaser*	*Salix arctica*	34	LRD50	2	-0.039	0.020	0.063	0.01	0.05	TEMP_JJA	1	0.034	0.011	0.003	0.01	0.05

Table S14. Linear mixed-effect models for double detrended series. Parameter estimates (β) with their associated 95% confidence intervals for linear mixed-effect (LME) models for *increasers* (top) and *decreasers* (bottom) fitted to each responder group using all shrubs, instead of chronologies for common period (1980-2008), and double detrended series (both RWI for each shrub and sea ice-related predictors time-series). A random intercept for a site was included in each model. For the source data and the abbreviations, see Table S9. SE - standard error; AICc - Akaike's information criteria with small sample bias adjustment; R^2m - marginal R^2; R^2c - conditional R^2. Not significant values are marked in grey italics. Note various number of df (i.e., pairs tested) within each model, as a results of AR detrending where some series were shortened. Therefore, model rank should be taken with caution.

increasers

model	Intercept	β	lower CI	upper CI	SE	df	t-value	sd: Site	sd: Residual	AICc	delta	weight	predictor p-value	Intercept p-value	R^2m	R^2c
pFAD	0.006	0.041	0.010	0.071	0.015	10215	2.6	0.000	0.980	26454	0	1	0.009	0.699	0.002	0.002
pSON	-0.003	-0.041	-0.072	-0.010	0.016	10354	-2.6	0.000	0.982	26822	369	0	0.010	0.864	0.002	0.002
pLRD	0.001	-0.019	-0.048	0.010	0.015	10423	-1.3	0.000	0.982	27008	555	0	0.199	0.958	0.000	0.000
DJF	0.004	-0.008	-0.037	0.021	0.015	10662	-0.5	0.000	0.979	27618	1164	0	0.587	0.797	0.000	0.000
pSON_pA	-0.005	-0.110	-0.139	-0.081	0.015	10692	-7.4	0.000	0.973	27625	1172	0	0.000	0.718	0.012	0.012
JJA_pA	-0.002	-0.074	-0.103	-0.046	0.015	10692	-5.1	0.000	0.976	27654	1200	0	0.000	0.885	0.006	0.006
LRD	0.01	-0.119	-0.147	-0.090	0.014	10708	-8.2	0.000	0.972	27670	1217	0	0.000	0.516	0.014	0.014
DJF_pA	0.002	0.012	-0.018	0.041	0.015	10692	0.8	0.000	0.979	27679	1226	0	0.431	0.899	0.000	0.000
JJA	-0.006	-0.099	-0.129	-0.070	0.015	10737	-6.7	0.000	0.975	27788	1335	0	0.000	0.708	0.009	0.009
MAM	0.004	0.013	-0.016	0.042	0.015	10730	0.9	0.000	0.981	27834	1381	0	0.384	0.811	0.000	0.000
MAM_pA	0.006	0.072	0.044	0.100	0.014	10821	5.0	0.000	0.979	28079	1625	0	0.000	0.679	0.005	0.005
null model	0.003	n.a.	-0.027	0.032	0.015	10822	0.2	0.000	0.982	28102	1648	0	n.a.	0.859	0.000	0.000

decreasers

model	Intercept	β	lower CI	upper CI	SE	df	t-value	sd: Site	sd: Residual	AICc	delta	weight	predictor p-value	Intercept p-value	R^2m	R^2c
pFAD	-0.007	0.023	-0.011	0.058	0.018	7105	1.3	0.000	0.966	18390	0	1	0.189	0.686	0.001	0.001
pLRD	0.001	-0.015	-0.048	0.017	0.017	7242	-0.9	0.000	0.972	18861	471	0	0.361	0.945	0.000	0.000
pSON	0.000	0.047	0.012	0.081	0.018	7309	2.7	0.000	0.974	19063	672	0	0.008	0.985	0.002	0.002
LRD	-0.001	0.008	-0.024	0.041	0.017	7459	0.5	0.000	0.979	19539	1149	0	0.617	0.953	0.000	0.000
JJA	0.005	0.079	0.046	0.112	0.017	7471	4.7	0.000	0.977	19559	1169	0	0.000	0.786	0.006	0.006
DJF_pA	0.006	0.095	0.061	0.129	0.017	7481	5.5	0.000	0.974	19601	1211	0	0.000	0.706	0.009	0.009
pSON_pA	0.006	0.089	0.055	0.123	0.017	7481	5.1	0.000	0.974	19605	1215	0	0.000	0.717	0.007	0.007
JJA_pA	0.002	0.006	-0.027	0.039	0.017	7481	0.4	0.000	0.978	19631	1241	0	0.714	0.907	0.000	0.000
MAM	0.001	0.038	0.006	0.071	0.017	7481	2.3	0.000	0.981	19650	1260	0	0.022	0.955	0.002	0.002
DJF	0.007	0.022	-0.012	0.055	0.017	7504	1.3	0.000	0.980	19714	1323	0	0.204	0.704	0.000	0.000
MAM_pA	0.008	0.109	0.077	0.142	0.017	7534	6.6	0.000	0.976	19772	1381	0	0.000	0.620	0.012	0.012
null model	0.002	n.a.	-0.032	0.036	0.017	7535	0.1	0.000	0.983	19813	1422	0	n.a.	0.887	0.000	0.000

SI References:

1. N. Boulanger-Lapointe, E. Lévesque, S. Boudreau, G. Henry, N. Schmidt, Population structure and dynamics of Arctic willow (Salix arctica) in the High Arctic. *J. Biogeogr.* **41**, 1967-1978, (2014).
2. P. Ropars, E. Lévesque, S. Boudreau, S. How do climate and topography influence the greening of the forest-tundra ecotone in northern Quebec? A dendrochronological analysis of Betula. *J. Ecol.* **103**, 679-690 (2015).
3. I. H. Myers-Smith *et al*., Climate sensitivity of shrub growth across the tundra biome. *Nat. Clim. Change* **5**, 887-891 (2015).
4. J. Hollesen, A. Buchwal, G. Rachlewicz, B. U. Hansen, M. O. Hansen, O. Stecher, B. Elberling, Winter warming as an important co-driver for Betula nana growth in Western Greenland during the past century. *Glob. Change Biol.* **21**, 2410-2423 (2015).
5. A. Buchwal, G. Rachlewicz, P. Fonti, P. Cherubini, H. Gärtner, Temperature modulates intra-plant growth of Salix polaris from a high Arctic site (Svalbard). *Polar Biol.* **36**, 1305-1318 (2013).
6. C. M. Gamm, P. F. Sullivan, A. Buchwal, R. J. Dial, A. B. Young, D. A. Watts, S. M. P. Cahoon, J. M. Welker, E. Post, Declining growth of deciduous shrubs in the warming climate of continental western Greenland. *J. Ecol.* **106**, 640-654 (2018).
7. D. Blok, U. Sass-Klaassen, G. Schaepman-Strub, M.M.P.D. Heijmans, P. Sauren, F. Berendse, What are the main climate drivers for shrub growth in Northeastern Siberian tundra? *Biogeosciences* **8**, 1169-1179 (2011).
8. M. Macias-Fauria, B. C. Forbes, P. Zetterberg, P., T. Kumpula, Eurasian Arctic greening reveals teleconnections and the potential for structurally novel ecosystems. *Nat. Clim. Change* **2**, 613-618 (2012).
9. A. Buchwal, S. Weijers, D. Blok, B. Elberling, Temperature sensitivity of willow dwarf shrub growth from two distinct High Arctic sites. *Inter. J. Biometeorol.* **63**, 167-181 (2019).
10. B. C. Forbes, M. Macias-Fauria, P. Zetterberg, P. Russian Arctic warming and `greening' are closely tracked by tundra shrub willows. *Glob. Change Biol.* **16**, 1542-1554 (2010).
11. P. F. Sullivan, A. Z. Csank, Contrasting sampling designs among archived datasets: implications for synthesis efforts. *Tree Physiol.* **36**, 1057-1059 (2016).
12. J. C. Stroeve, A. D. Crawford, S. Stammerjohn, Using timing of ice retreat to predict timing of fall freeze-up in the Arctic. *Geophys. Res. Lett.* **43**, 6332-6340 (2016).
13. S. M. Vicente-Serrano, S. Beguería, J. I. López-Moreno, A Multi-scalar drought index sensitive to global warming: The Standardized Precipitation Evapotranspiration Index - SPEI. *J. Clim.* **23**, 1696 (2010).
14. S. Beguería, S. M. Vicente-Serrano, SPEI: Calculation of the standardised precipitation-evapotranspiration index. Version 1.7, R package. http://cran.r-project.org/web/packages/SPEI/index.html (2017).
