# Supplementary material for: Divergence of Arctic shrub growth associated with sea ice decline
Source: Proc Natl Acad Sci U S A. 2020 Dec 14;117(52):33334–44. doi: 10.1073/pnas.2013311117 (PMC7776857; doi:10.1073/pnas.2013311117)
Supplement: Supplementary File [file pnas.2013311117.sd01.pdf]

```
### This file presents an example code used for the analyses performed in the ICY SHRUB
synthesis:
### Buchwal et al. (26 June 2020 - submission date):
### Article Title: Divergence of Arctic shrub growth associated with sea ice decline



install.packages("dplR")
library(dplR)
install.packages("treeclim")
library(treeclim)

install.packages("nlme")
library(nlme)
install.packages("MuMIn")
library(MuMIn)
install.packages("piecewiseSEM", dependieces=T)
library(piecewiseSEM)



#######################################################################
###
### Fig. 2
###
### Moving correlation analyses relating mean increasers (example shown below) and
decreasers
### chronologies to monthly sea-ice extent (SIE) for the Arctic Ocean

B<-dcc(ALL_CRN_dRCS_NEG.crn[93:121,], SIE_dcc[1:29,], selection=-6:8,
method="correlation", dynamic = "moving", win_size=20,
win_offset=1, ci=0.05,var_names="SIE",boot="std")

plot(B)


# variables used:
# ALL_CRN_dRCS_NEG.crn = mean responder-type chronology for increasers (arithemtic mean
computed for 13 individual detrended (dRCS) chronologies, all increasers)
# SIE_dcc = Pan-Arctic sea ice extent (SIE) in monthly resolution (from January to
December)



#######################################################################
###
### Fig. 5A, 5B  & Table S10 and Table S11
###
### Piecewise structure equation models (SEM) for each responder group (example for
decreasers shown)
### A random intercept for a site (ID.s) was included in each linear mixed-effect (LME)
model

psem_POSb <- psem(
  lme(MRW_dRCS~LRD50_Z + TEMP_JJA_Z + SPEI_JJA, random=list(ID.s=~1), data =
MYDATA_POS_CRN_ar, na.action = na.omit),
  lme(SPEI_JJA~LRD50_Z  + PRECIP_Z_JJA + TEMP_JJA_Z, random=list(ID.s=~1), data =
MYDATA_POS_CRN_ar, na.action = na.omit),
  lme(PRECIP_Z_JJA~LRD50_Z  + TEMP_JJA_Z, random=list(ID.s=~1), data =
```

```
MYDATA_POS_CRN_ar, na.action = na.omit),
  lme(TEMP_JJA_Z~LRD50_Z, random=list(ID.s=~1), data = MYDATA_POS_CRN_ar, na.action =
na.omit)
 )

POS_LRD50_Z  <- summary(psem_POSb)
AIC(psem_POSb, aicc=TRUE)


# variables used:
# MRW_dRCS = mean ring width after dRCS detrending for each species-by-site chronology
# LRD50_Z = last retreat day of sea ice at the 50% of sea ice concentration threshold
(z-score)
# TEMP_JJA_Z = June-July-August mean temperature (z-score)
# SPEI_JJA = June-July-August mean standardized precipitation evaporation index
# PRECIP_Z_JJA = June-July-August precipitation sum (z-score)
# ID.s = species-by-site distinction
# MYDATA_POS_CRN_ar = data frame with all detrended mean annual growth records for 9
decreasers chronologies and standardized sea ice and climate variables



########################################################################
###
### Table S12.
###
### linear mixed-effect (LME) models for increasers (as an example shown).
### A random intercept for a shrub (Shrub) nested within a site (Site.G) was included in
each model

m3_pSON_pA<-lme(RWIsq~pSON_pA, random=list(~1|Site.G, ~1|Shrub), data = MYDATA_NEG_aS,
method="ML",
                        cor=corAR1(), control=list(maxIter=1000),na.action = na.exclude)
summary(m3_pSON_pA)
r.squaredGLMM(m3_pSON_pA)


## null model:
m3_mNULL<-lme(RWIsq~1, random=list(~1|Site.G,  ~1|Shrub), data = MYDATA_NEG_aS,
method="ML",
                        cor=corAR1(), control=list(maxIter=1000),na.action = na.exclude)
summary(m3_mNULL)
r.squaredGLMM(m3_mNULL)


### DIAGNOSTIC PLOTs

plot(m3_pSON_pA)

# 1: to check normal distribution of random effects:
plot(ranef(m3_pSON_pA, level=1))   # check level=1 and level=3 for each random effect

# 2: to check homogeneity of variance:
plot(fitted(m3_pSON_pA), residuals(m3_pSON_pA), xlab = "Fitted Values", ylab =
"Residuals")
abline(h = 0, lty = 2)
lines(smooth.spline(fitted(m3_pSON_pA), residuals(m3_pSON_pA)))

# 3: to check if resuduals of the model are normally distributed:
```

```
require("lattice")

qqnorm(resid(m3_pSON_pA))
qqline(resid(m3_pSON_pA), col="red", lty=2, lwd=2)

hist(resid(m3_pSON_pA))
d<-density(resid(m3_pSON_pA), na.rm = TRUE)
plot(d)

hist(resid(m3_pSON_pA))
hist(resid(m3_pSON_pA), breaks=100)

mean(resid(m3_pSON_pA))

# 4: checking for linearity
Plot.Model.F.Linearity<-plot(resid(m3_pSON_pA),MYDATA_NEG_aS_ar$RWIsq)


# variables used:
# RWIsq = all shrubs mean ring width index (RWI) after dRCS detrending and square root transformation
# pSON_pA = previous September-October-November mean Pan-Arctic sea-ice extent (z-score)
# MYDATA_NEG_aS = data frame with all detrended mean annual growth records for all shrubs assigned to increasers with sea ice variables



#######################################################################
###
### Table S13.
###
### linear mixed-effect (LME) models for each spiecies-by-site chronology (Site.G) run for each sea ice variable
### (here for Pan-Arctic autumn (from pervious September to previous November) sea ice extent as an example).
### A random intercept for a year was included in each model

MYDATA_DD_aS<-as.data.frame(MYDATA_DD_aS)

colnames(MYDATA_DD_aS)

# order by alphabetic:

MYDATA_DD_aS<-with(MYDATA_DD_aS,MYDATA_DD_aS[order(Site.G) , ])

head(MYDATA_DD_aS)
tail(MYDATA_DD_aS)


GROUPS <- unique(MYDATA_DD_aS$Site.G)

for (i in 1:length(GROUPS)){
  CURRENT_GROUP <- GROUPS[i]

  df <- filter(MYDATA_DD_aS, Site.G == CURRENT_GROUP)   # subset the dataframe
  fit <- lme(RWIsq~pSON_pA, random= ~1|YEAR, data = df, method="ML", cor=corAR1(), control=list(maxIter=1000),na.action = na.exclude)
  print(summary(fit)$tTable)
  #print(summary(fit)$AIC)
```

```
  #print(r.squaredGLMM(fit))
  #print(AICc(fit))
 }

#str(summary(fit))


## NULL model:   # random=~1|YEAR

GROUPS <- unique(MYDATA_DD_aS$Site.G)

for (i in 1:length(GROUPS)){
  CURRENT_GROUP <- GROUPS[i]

  df <- filter(MYDATA_DD_aS, Site.G == CURRENT_GROUP)   # subset the dataframe
  fit <- lme(RWIsq~1, random= ~1|YEAR, data = df, method="ML", cor=corAR1(),
control=list(maxIter=1000),na.action = na.exclude)
  #print(summary(fit)$tTable)
  AIC_null<-print(summary(fit)$AIC)
 }


# variables used:
# RWIsq = all shrubs mean ring width index (RWI) after dRCS detrending and square root
transformation
# pSON_pA = previous September-October-November mean Pan-Arctic sea-ice extent (z-score)
# MYDATA_DD_aS = data frame with all detrended mean annual growth records for all shrubs
and standardized climate variables



########################################################################
###
### Table S14.
###
### linear mixed-effect (LME) models for increasers (as an example) and Pan-Arctic pSON
SIE
### and double detrended series (both RWI for each shrub and sea ice-related
### predictors time-series). A random intercept for a site (Site.G) was included in each
model.

m3_pSON_pA<-lme(RWIar~pSON_pA_DD, random=list(~1|Site.G), data = MYDATA_NEG_aS_ar,
method="ML",
                        cor=corAR1(), control=list(maxIter=1000),na.action = na.exclude)
summary(m3_pSON_pA)
intervals(m3_pSON_pA, which="fixed", level = 0.95)
r.squaredGLMM(m3_pSON_pA)


# variables used:
# RWIar = detrended shrub growth (for each individual shrub) after first dRCS and then
AR detrening
# pSON_pA_DD = Pan-Arctic autumn (i.e., from previous September to previous November)
sea ice extent after AR detrending
# MYDATA_NEG_aS_ar = data frame with all double detrended mean annual growth records for
all shrubs and detrended sea ice variables
```

```
########################################################################
###
### example for climate data standardization (z-score) at a site level:

TEMP_JJA_Z<-scale(TEMP_JJA[,-1],center=T)


# variables used:
# TEMP_JJA = raw June-July-August mean temperature for particular site for the common
period (1979-2008)
# TEMP_JJA_Z = standardized June-July-August mean temperature for particular site



########################################################################
###
### example for AR detrending for regional sea ice extent:

REG_SIEw_AR<-detrend(REG_SIE_window_RWL,make.plot=TRUE, method="Ar")


# variables used:
# REG_SIE_window_RWL = rwl file for regional sea ice variables in seasonal resolution
for each site for the common period (1979-2008)
# REG_SIEw_AR = object with detrended (Ar) regional sea ice variables in seasonal
resolution for each site
```
